# Dynamics of task preparation processes revealed by effect course analysis on response times and error rates

**DOI:** 10.1038/s41598-024-54823-1

**Published:** 2024-02-21

**Authors:** Alexander Berger, Wilfried Kunde, Markus Kiefer

**Affiliations:** 1 Department of Psychiatry, Section for Cognitive Electrophysiology, Ulm University, Leimgrubenweg 12, 89075 Ulm, Germany; 2 Department of Psychology, University of Würzburg, Würzburg, Germany

**Keywords:** Psychology, Human behaviour

## Abstract

Cuing or executing a task impacts processing pathways for task-relevant information. While there is ample evidence that processing associated with task execution changes with practice, such evidence regarding cue-induced task preparation is scarce. Here we explored practice-related changes of processing pathways by task cuing in order to assess the plasticity of task preparation. We first developed and validated a new method for the study of practice-related changes, the effect course analysis. The effect course analysis is a model-free, non-parametric method designed to reveal effect changes within an experimental session on a continuous time scale. Then we applied this method to a new study in which cued task sets were supposed to remain activated during assessment of task-relevant pathways, as potential task execution was postponed at the end of the trial. The results showed that, with little practice, task cuing amplified task-relevant pathways, whereas this effect vanished with practice, suggesting that practice prompts fundamental changes of how task cues are used for task preparation. Hence, if one cannot be certain that cognitive processing is stationary, investigating the time course of experimental effects appears to be crucial to determine how cognitive processing is influenced by practice.

## Introduction

Human operators are remarkably capable in adjusting cognitive processing according to changing task demands; however such flexible changes in cognitive operations, i.e. like in switching between different tasks^1^, are often associated with costs in terms of slower or more erroneous responses^2,3^. The flexibility of the cognitive system can be, for instance, studied within the so-called task switching paradigm^2^. In this paradigm, participants have to perform at least two different tasks, while performance is usually impaired when the task switches compared to when it is repeated, referred to as switch costs^4,5^. Furthermore, research in task switching showed that switch costs can be reduced, if more time is available to prepare for a task switch^5–7^, suggesting a crucial role of task preparation for the optimization of task performance.

Traditional data analysis strategies in cognitive psychology including the field of cognitive flexibility and task switching aggregate performance measures such as response times or errors condition-wise across a large series of experimental trials to ensure a sufficient signal-to-noise ratio of the dependent measure. This analysis strategy rests on the tacit critical assumption that cognitive processes, e.g. processes underlying task preparation, are stationary, i.e. do not change during the aggregated series of trials. However, analyses demonstrating that several components of the drift–diffusion model, representing different cognitive processes^8,9^, change during the course of an experiment^10–12^, as well as the observation that performance at different time points of a working memory task is differentially predictive for fluid intelligence^13^, challenge this assumption of stationarity. Such changes in cognitive processing with practice can be subsumed under the account of the *plasticity* of the cognitive system, which can be described as an ongoing modification of cognitive processes as a function of task practice^2,14^. Such a plasticity of cognitive processing is widely accepted regarding task processing, for example reflected by an overall decrease of response times during an experiment, or more specifically, by a facilitated switching between tasks with more practice^2,15^. However, concerning the plasticity of task preparation processes, research is lacking. Therefore, the aim of the present study is to demonstrate that also task preparation processes are plastically modified.

To investigate potential plasticity of task preparation, task cues without subsequent task execution (*task cue-only*) can be presented^16,17^. Task cues serve to indicate the identity of the to-be-performed task on a trial-by-trial basis^18^ and the processing of task cues is considered a proactive control process^19,20^: when presented with task cues, participants can activate the task set in advance, that is the cognitive configuration required for executing the task^1,4^, facilitating subsequent execution of the cued task^5–7^. Besides such strategic components of task cue processing, studies on task cue priming indicated that task sets are also triggered by masked, and therefore only unconsciously processed, task cues^21–23^. Hence, the association of task cue and task set might be strong enough, that a task cue can automatically prime or retrieve the associated task set.

According to the attentional sensitization model of unconscious cognition^24^, the activation of task sets following a task cue-only may influence subsequent unconscious processing in terms of a sensitization or de-sensitization of corresponding processing pathways. For example, if a semantic task, like categorizing pictures into living and non-living objects, is performed before assessing masked semantic priming in a lexical decision task (LDT), the activated semantic task set sensitizes processing pathways involved in semantic prime processing, thereby enhancing semantic priming^24^. Conversely, an activated perceptual task set, like categorizing pictures into round and elongated objects, enhances prime processing in perceptual pathways, but reduces prime processing in semantic pathways and thus masked semantic priming. Semantic priming is defined as a faster and less error prone response to a target word, if it is preceded by a semantically related (e.g. table–chair) compared to an unrelated prime word (e.g. car–sky) and can also be observed, if prime words are masked and therefore only unconsciously processed^25^. This phenomenon, i.e. larger masked semantic priming following a semantic compared to a perceptual task, could be repeatedly demonstrated^16,17,24,26–29^.

Furthermore, mere presentation of task cues have been shown to activate task sets by differentially sensitizing semantic processing pathways. If a task cue triggers a semantic task set, subsequent semantic priming should be enhanced. In contrast, if the semantic task set is suppressed following task cue presentation, for instance because it is not currently required^16,17^, semantic priming should be attenuated, as a suppression of task sets should result in a de-sensitization of associated processing pathways^16,24^. Hence, the modulation of masked priming subsequent to the presentation of a task cue should be indicative for a cue-induced activation of task sets and therefore for the engagement into task preparation. This influence of cued tasks sets on subsequent priming might also change during the course of the experiment, as we previously found signs of an increase of cue-related modulations of masked semantic priming as a function of practice^17^.

The present study therefore further investigated the plasticity of task preparation processes. In this experiment, we presented task cue-onlies, followed by a masked primed LDT, in order to assess how cued task sets modulate subsequent priming. Task cues indicated either a semantic or a perceptual task (so-called *induction tasks*) and should therefore enhance or attenuate subsequent semantic priming, respectively. To ensure that participants do not ignore the task cues, we postponed execution of cued task sets after the LDT, and participants should therefore be required to process the task cue in order to be able to later apply the cued task set, if an induction task is presented. Previous work in other contexts already showed that cueing a task, which is only required later, impacts performance in a subsequent, different task^30,31^, see also^32^. Furthermore, effects of task cues in this context also changed with practice^33^.

Previous investigations on the influence of practice and according changes in response times (RTs) and error rates (ERs) in cognitive psychology typically divided the data into subsets of trials (i.e. blocks) and aggregated the data separately per block. However, there is a large variability between studies with regard to number of trials per block and total number of trials. For instance, in by ourselves arbitrarily selected studies the number of trials per block had a range of 66–240, with total trial numbers ranging from 720 to > 23,000 trials^34–37^. However, changes in experimental effects may even occur on a more fine-grained time scale, within tens or a couple of trials, for which such an analysis depending on block number appears to be of only limited use. Accordingly, we suggest that the time course of an effect across an experimental session needs to be studied on a continuous scale to detect such fine-grained changes. Furthermore, when the time course of an effect is modelled by a regression analysis on single trial data, the assumed type of the change has to be specified a-priori (or tested using model comparison). Similar, approaches studying the development of experimental effects using drift–diffusion modelling depend on an underlying model structure^10,11,38^.

Hence, within the context of the current study, we present a new non-parametric and model-free method to analyze performance changes across time at a relatively high temporal resolution—the *effect course analysis*—which does not require assumptions about the type of the modulation over time. To account for the large variability of single-trial data^39,40^, this method uses smoothed RTs and ERs calculated by moving averages, which are a widely used tool to reduce the influence of short-term fluctuations for example in economics^41,42^. Then, the moving averages of two experimental conditions are statistically compared. To assess whether the difference between experimental conditions is reliable over time, i.e. extends over several trials, non-parametrical cluster testing is applied. Such cluster-based permutation tests (CBPT) are already established in the analysis of high-dimensional neurophysiological (EEG/MEG) as well as neuroimaging data^43–45^. Following this procedure, an effect is only considered significant, if it extends over several trials and it is unlikely to obtain such an effect by chance (i.e. in random partitions of the data). For a more detailed description of the effect course analysis used in the present work, see the “Method” section.

To summarize, the aim of the present study is to apply a new method for analyzing changes of experimental effects during the course of one experimental session to investigate the plasticity of task cue effects on masked priming. In Study 1, we firstly validated the proposed effect course analysis on data of the mega-study of Adelman and colleagues^46^, for which qualitative different time courses for two effects were already described^15^. A word/nonword effect was present during the whole session, but initially increased until it reached its maximum and further started to decrease. A response repetition effect was initially absent and could only be observed after a couple of trials, but then started to progressively increase. The effect course analysis is supposed to recover these two previously described distinct time courses (for subtle deviations according to methodological differences, see the “Discussion”).

We then apply the effect course analysis to reveal fine-grained changes of task cue processing (Study 2, for which the effect course analysis was pre-registered) as indexed by a change in attentional sensitization of subsequent masked priming. In this experiment, task cue-onlies were presented, which cued either a semantic or perceptual task set, followed by an assessment of semantic priming in a LDT to be able to infer from the (de-)sensitization of priming the activation of cued task sets. Execution of cued task sets was postponed after the LDT to ensure an appropriate need of task cue processing. We hypothesized two possible effects of task cues on masked semantic priming in the LDT:

First, as cued task sets may be required later in the postponed induction task, they are kept activated throughout the LDT. Cued task sets accordingly sensitize priming in terms of larger semantic priming following a task cue associated with the semantic task. As our previous work indicated the strength of prepared task sets to increase with practice^17^, this sensitization of semantic priming following the semantic task set should increase during the experiment. Second, despite the need of later execution of cued task sets, keeping cued task sets activated interferes with the different, lexical task set of the LDT. Therefore, cued task sets become suppressed (and supposedly re-activated later after the LDT). A suppression of task sets should result in a de-sensitization of associated processing pathways and accordingly in larger semantic priming following the perceptual task set. Similar to the first hypothesis, the amount of interference and therefore the size of the priming modulation by task sets should increase during the course of the experiment. To foreshadow the results, the result pattern was more in line with the first hypothesis. However, it did not indicate a gradual, but a qualitative change in processing, i.e. which kind of information (task set vs. task identity) was kept active during the LDT appeared to change during the experiment.

## Results

### Study 1: re-analysis of data from Adelman et al. (2014)

Study 1 aimed to demonstrate that the proposed effect course analysis succeeds in recovering previously described effect courses. To this end, we re-analyzed two effects of a study by Adelman and colleagues^46^, which was designed to investigate masked form priming^47^ with a variety of different primes. Participants had to classify targets in a lexical decision task as word or nonword, which were preceded by a masked prime. We chose to re-analyze the word/nonword effect as well as a response repetition effect according to their previously described different time courses^15^. The word/nonword effect (slower responses for nonword than word targets) was large and present from the beginning of the experiment and initially increased further until it reached its maximum around approximately 40–50 trials and then started to decrease again. The response repetition effect (faster responses for response repetitions than switches) was small and initially absent, but increased progressively after a few trials. These two qualitative different effect courses should serve to test whether the effect course analysis is able to reproduce different shapes of effect courses.

#### Effect course analysis of the word/nonword effect

**Figure 1:**
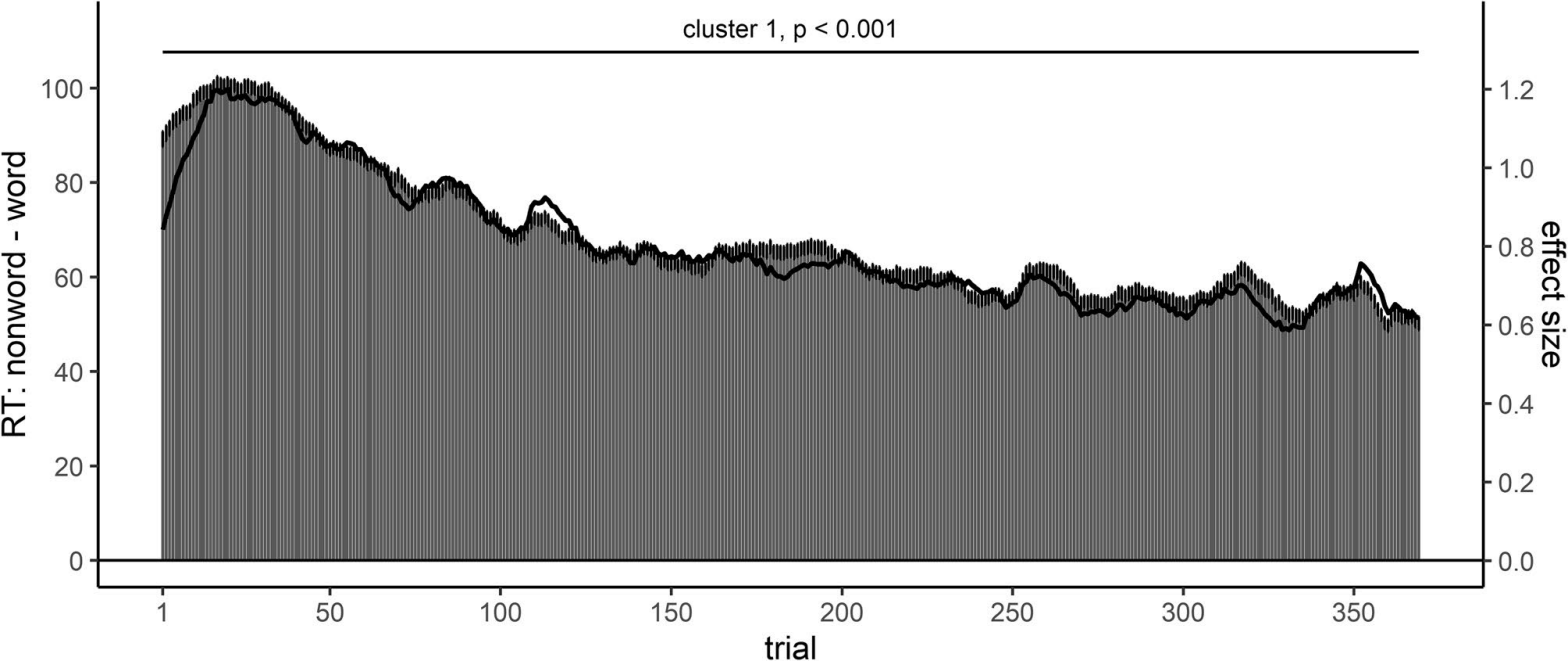
Effect course analysis of RTs for the word/nonword effect in the Adelman et al. (2014) study. Positive values indicate slower responses for nonword compared to word targets. In this and the upcoming figures, the bars show the (moving averaged) RT difference at each trial (scale at the left side) plus standard errors, while the solid line shows the standardized effect size of the difference at each trial (scale at the right side).

Effect course analysis for the word/nonword effect revealed one significant cluster, spanning across the whole analyzed time window (trial 1—369), *T* = 9323.00, *p* < 0.001. For a graphical depiction of the effect course, see Fig. 1 (for corresponding figures for ERs, see Supplementary Material C), which also revealed no remarkable difference between raw (RT difference) and standardized effect size. This displayed effect course could recover previously reported practice-induced changes of this effect^15^: the effect initially increased until reaching its maximum between 20 and 40 trials with a maximum effect of around 100 ms, and subsequently decreased.

#### Effect course analysis of the response repetition effect

**Figure 2:**
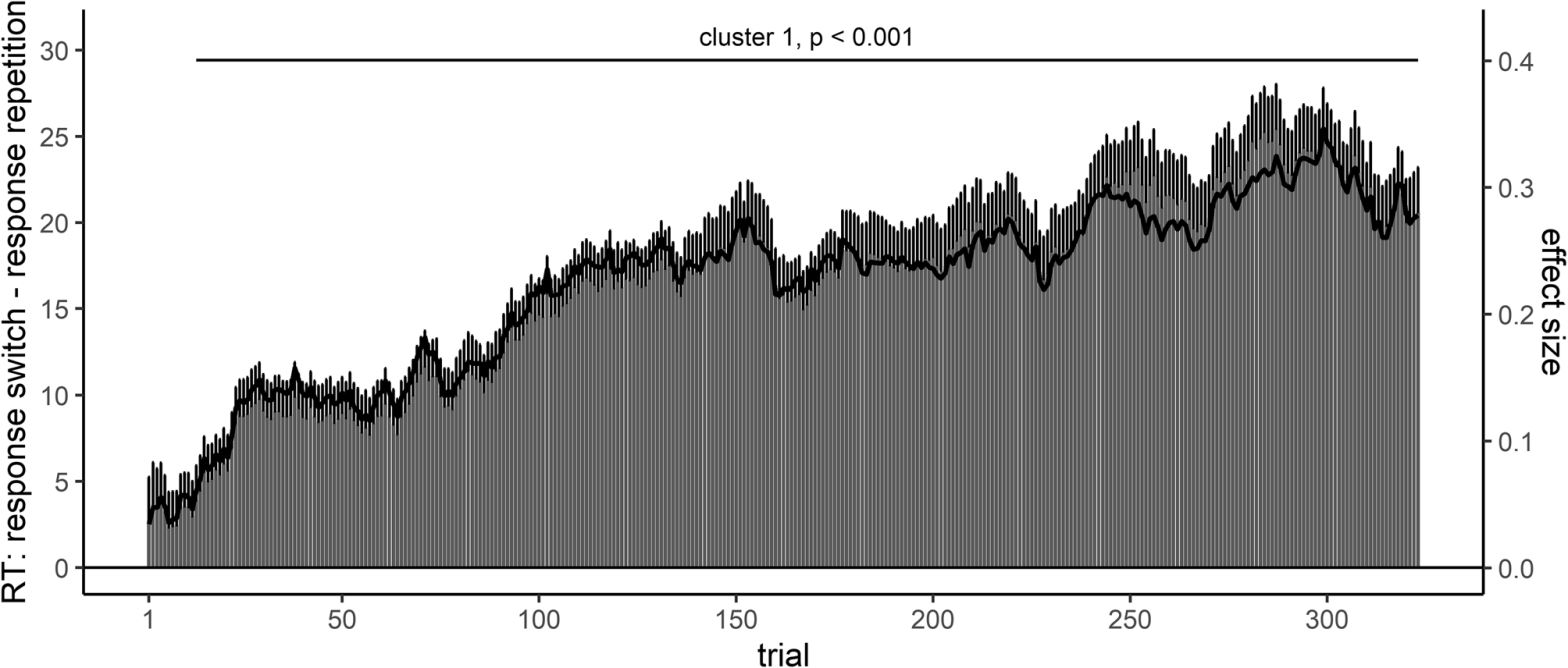
Effect course analysis of RTs for the response repetition effect in the Adelman et al. (2014) study. Positive values indicate slower responses for response switches compared to response repetitions.

The effect course analysis of RTs for the response repetition effect revealed one significant cluster, *T* = 2222.70, *p* < 0.001, which however did not include the first trials, see Fig. 2. The cluster spanned from trials 13 to 323 (i.e., the end of the analyzed time window). Accordingly, effect course analysis could also recover the previously described initial lack and subsequent increase of this effect^15^, which was comparable for both the mean difference as well as the standardized effect size.

### Study 2

In Study 2 we aimed to demonstrate that also task preparation processes are plastically modified during the course of one experimental session. In this study, task cues instructing a semantic or perceptual task were followed by a masked primed LDT in order to assess how activation of task sets following task cue presentation modulates subsequent semantic priming as index of cue-induced task set activation. Execution of cued task sets in induction tasks was postponed after the LDT, resulting in two trial types, which were presented intermixed and randomly:

Induction task trial: Task Cue—masked primed LDT—Induction task.

Task cue-only trial: Task Cue—masked primed LDT.

In both trial types, the procedure until the end of the LDT was identical and they were therefore collapsed for statistical analysis. The presentation of the postponed induction task in induction task trials served to ensure that participants are required to process the task cues, as they may have to execute the cued task set later. Induction tasks were only presented in half of the trials (induction task trials and task cue-only trials were presented equally often) to ensure comparability to our previous work^16,17^, which indicated a suppression of cued task sets in task cue-only trials, when it was evident that the cued task set is no longer required (in these studies, induction tasks in induction task trials were immediately presented after the task cue and the lack of an induction task in task cue-only trials was therefore evident with onset of the LDT).

Study 2 investigated the plasticity of task preparation processes in terms of how task cue processing as instance of task preparation modulates subsequent priming. According to the attentional sensitization model^24^, an activation of task sets following task cue presentation should sensitize priming in terms of larger semantic priming following a semantic compared to a perceptual task set. Therefore, we used the effect course analysis to contrast, how masked semantic priming differs following task cues associated with the semantic versus the perceptual task set. Beforehand, to establish that differences in the modulation of priming by cued task sets were not a mere consequence of the effect course of the raw priming effect, we first performed an effect course analysis on the priming effect collapsed across task sets. For example, the modulation of priming by task sets could simply lack at the end of the experiment, if there is no reliable priming at all later during the experiment. However, effect course analysis of the priming effect collapsed across task sets did not support such a simple explanation (for corresponding figures, see Supplementary Material B). RT priming was consistently observed during the whole experiment, which was reflected by a corresponding cluster, *T* = − 592.58, *p* < 0.001, trials 1–141. Similarly, there were no hints that ER priming qualitatively changed throughout the experiment. A corresponding cluster extended over almost the whole duration of the experiment, *T* = -522.45, *p* < 0.001, trials 7–150.

#### Effect course analysis of the modulation of priming by cued task sets

**Figure 3:**
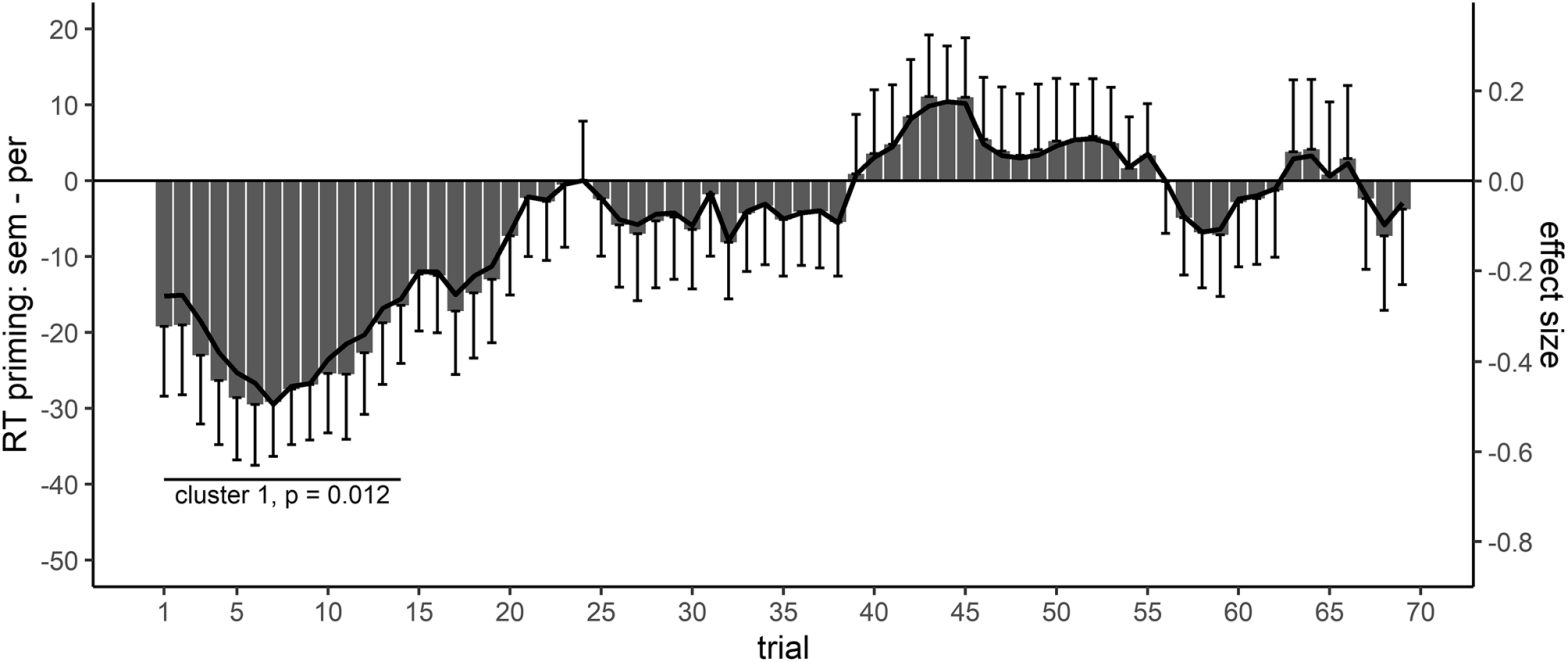
Effect course analysis for RTs in Study 2. Negative values indicate larger semantic priming following task cues associated with a semantic compared to a perceptual task set. Only at the beginning of the experiment, a significant cluster reflected a modulation of priming by task cue-onlys, which vanished after a few trials.

Effect course analysis for the modulation of priming by cued task sets in Study 2 revealed one significant cluster for the analysis of RTs. Only at the beginning of the experiment, RT priming was larger for semantic than perceptual task sets, see Fig. 3, while later during the experiment no significant cluster associated with a modulation of priming by task sets could be observed. The time courses of the raw effect and of the standardized effect size were almost identical. CBPT showed the observed difference at the beginning of the experiment to be significant, *T* = − 41.84, *p* = 0.012, trials 1–14. Hence, in contrast to our hypotheses, the modulation of priming by task cue-onlys did not increase, but lacked after an initial effect later during the experiment. In Study 2, the strength of prepared task sets therefore did not appear to increase with practice as previously observed^17^, which might reflect a practice-induced qualitative change in task cue processing (see the “Discussion”). For ERs, no significant cluster could be observed, all *p*s > 0.235.

#### Influence of task practice prior to the start of the experiment

To ensure that participants were familiar with the experimental procedure upon the start of the main experiment in Study 2, all participants practiced the experimental procedure beforehand. Although all participants achieved a decent performance at the end of the practice block, there were however inter-individual differences between participants’ performance in the practice block. As such differences in task practice prior to the start of the main experiment may influence the plasticity of task preparation effects, we analyzed whether the observed modulation of priming described above differed as a function of participants’ performance in the practice block. To this end, we performed a median split according to participants’ performance in the practice block. Participants with an equal or higher proportion of errors than the sample’s median were considered to have a “bad” practice performance (n = 34), while the rest of participants were considered to have a “good” practice performance (n = 33). Afterwards, we calculated the above described effect course analysis separately for these two groups. Note that these analyses were not pre-registered and should therefore be considered exploratory.

**Figure 4:**
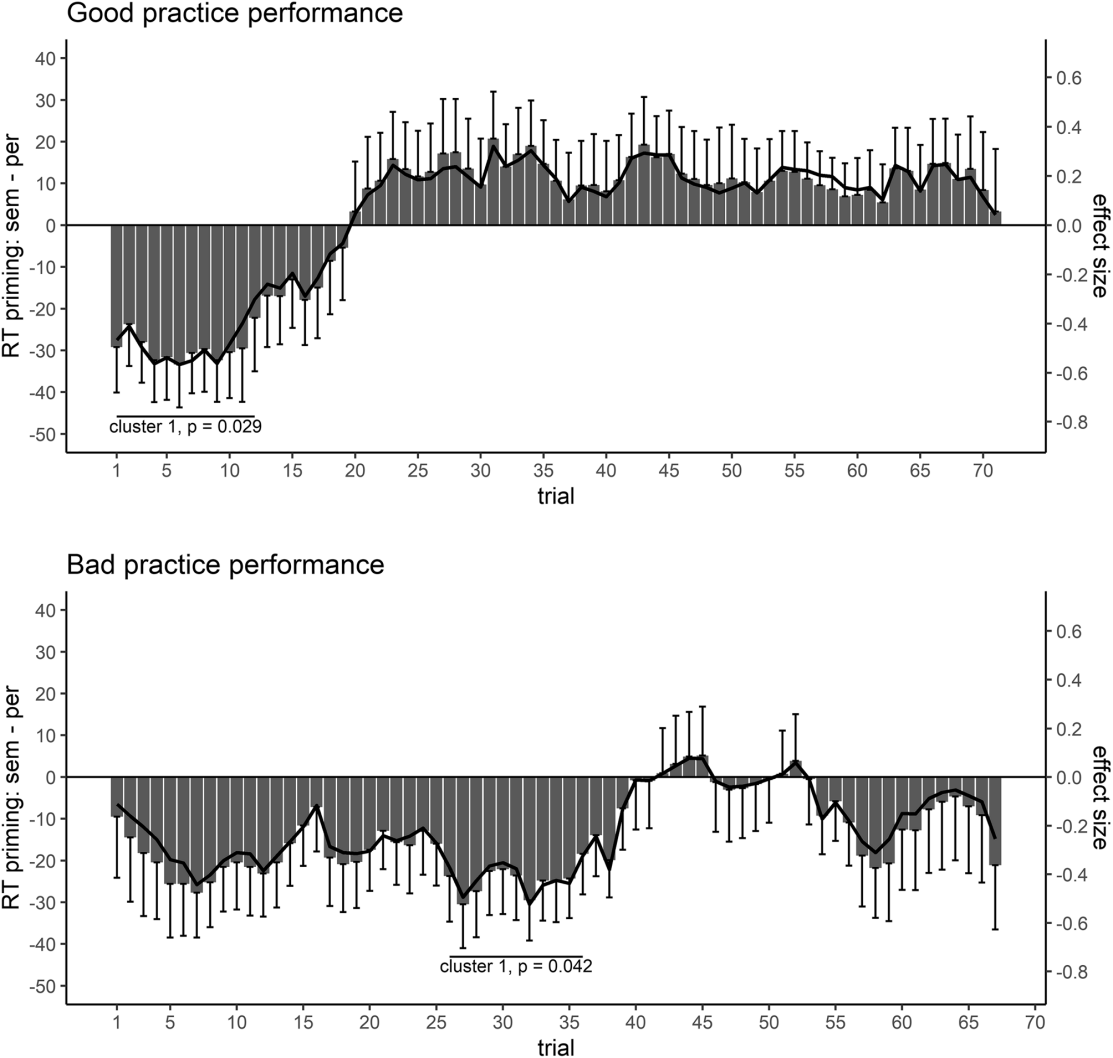
Effect course analysis for RTs in Study 2 depending on practice performance. The effect course of participants with good practice performance is shown in the upper panel, that one for participants with bad practice performance in the lower panel. Only significant clusters are shown.

For the analysis of RTs, the effect course analysis for the modulation of masked semantic priming by cued task sets revealed one significant cluster for the group with good practice performance. Masked semantic priming was larger for semantic than perceptual task sets only at the beginning of the experiment, reflected by a respective significant cluster, *T* = − 33.60, *p* = 0.029, trials 1–12. For the group with bad practice performance, a corresponding effect course analysis revealed three clusters indicating larger semantic priming for semantic task sets, of which only one cluster reached significance. The first cluster spanned from trials 5–13 (*T* = − 18.77, *p* = 0.081), the second one from trials 18–20 (*T* = − 5.42, *p* = 0.298) and the third one from trials 26–36, which reached significance (*T* = − 26.49, *p* = 0.042). The effect course analyses for both groups is shown Fig. 4. Corresponding effect course analysis for ERs revealed in neither of two groups any significant cluster.

When comparing the separate effect course analyses depending on the performance in the practice block, a similar initial presence and later absence of the modulation of priming by cued task sets was observable. However, while this modulation rapidly vanished in the group with good practice performance and was no longer observable after around a fifth of the experiment, this modulation lasted longer in the group with bad practice performance. Furthermore, in the group with bad practice performance, the modulation was only associated with a significant cluster around the middle of the experiment, indicating that the effect took some additional practice time to stabilize. Hence, while the sensitization of semantic priming by semantic task sets appeared to be stronger when practice performance was good, it also vanished faster. In contrast, if practice performance was bad, this modulation appeared to be somehow weaker, but more sustainable.

## Discussion

The present study proposed a new effect course analysis to continuously track the time course of an effect on a fine-grained time scale throughout an experimental session. This analysis method was first validated in Study 1 on an existing dataset^46^, for which changes of effects during the course of an experiment were already described^15^. The new effect course analysis recovered the previously described changes across trials. Second, by applying the effect course analysis to our own experiment, Study 2, it revealed rapid changes in task cue effects on priming, indicating task preparation effects triggered by task cues to vanish during an experimental session. Furthermore, the time course of this effect depended on the level of task practice prior to the start of the experiment. This highlights the importance for investigating the plasticity of cognitive processing.

Regarding the first goal of the present study, to establish that the proposed effect course analysis is able to recover previously described effect changes with practice, we re-analyzed two effects in the dataset of Adelman et al. (2014)^46^, for which Miller^15^ described different effect courses across trials; a word/nonword and a response repetition effect. The effect course analysis succeeded at recovering the shape of the effect course for both effects: the word/nonword effect was present from the beginning of the experiment and large. It increased until it reached a maximum effect of around 100 ms and then started to decrease. The response repetition effect was initially absent, but then started to progressively increase. While these two patterns could be recovered, there are however subtle differences between the current analysis and the analysis by Miller^15^, which can be traced back to methodological differences between both analysis approaches.

First of all, Miller investigated how statistical power can be maximized and therefore analyzed only subsets of the mega-studies’ data. Hence, the described effect changes with practice included fewer trial numbers compared to our analyses. Nevertheless, we considered it natural, when investigating the time course of an effect, to describe this course throughout the whole experiment, therefore including all trials into analysis. Accordingly, we can only compare the effect course for the first 200 (condition-wise) trials with data from Miller, that is approximately half of the experiment. However, the slope of the effect courses did not appear to change in the second half of the experiment, and a further comparison therefore would likely add only little information.

Second, and the most crucial difference in our view, as Miller varied the number of included trials by progressively including more trials (and accordingly less participants, as he was interested in which combinations of trial numbers and sample size could maximize statistical power), the reported practice-related changes reflect the comparison of *cumulative moving averages* in contrast to the reported *moving averages* in the present work. As a consequence, the effect course in the present work were shifted to the left compared to those reported in Miller^15^. For example, the peak of the word/nonword effect was around 40 trials using cumulative moving averages, while it was around 25 trials in the present analysis. To elaborate on this a bit more, using *cumulative* moving averages, the influence of all previous trials is carried onto the later averages thereby delaying the effect’s peak in the present example, as a larger number of trials is required to overcome this persisting influence of the previous trials. In contrast, the proposed effect course analysis uses the same number of trials in each moving average (here: 17; despite for the beginning/end of an experiment, where only fewer trials were available). We suppose moving averages therefore to be the more natural approach for investigating effect courses, as one does not need to consider how the influence of previous trials affects the shape of the effect course.

Last, considering the re-analysis of the response repetition effect, there were remaining small differences in the effect course between the present analysis and the one described by Miller^15^, which cannot be explained by the calculation of moving averages. For example, Miller showed the effect to be totally absent when using small trial numbers (effect of 0 ms for up to around 20–30 trials), while in the here described analysis an initial effect of around 5 ms was present. This was a consequence of a different data pre-processing between both studies. When changing the performed pre-processing steps, we could re-produce the previously described effect course. The relevance of data pre-processing for the inspection of effect courses was further highlighted by a total lack of the response repetition effect, if incorrect trials were not excluded from analysis (see Supplementary Material D). Accordingly, following a similar logic as it is usual assumed in traditional analysis strategies of RTs/ERs^48–50^, the exclusion of trials which likely do not represent the cognitive phenomena of interest seems to be crucial before conducting an effect course analysis. Following this line of reasoning, the interpretation of the temporal position of the obtained clusters within the experimental session should not be considered in terms of the presented trial in the experiment itself, but in terms of a relative position within the trials of the experiment. As some trials were excluded before performing the effect course analysis and the effect course was averaged across the trials of different participants, the given trial numbers naturally do not reflect the trial indices occurring for the particular participants.

As outlined above, the effect course analysis was applied on only a subset of all available data and therefore, the sequence of analyzed trials was not identical to the exact sequence of presented trials within the experiment. However, this does not compromise the interpretation of the effect course analysis, as long as the relative temporal ordering of the available per-condition trials is maintained, i.e. trials occurring at the beginning of the experiment remained at the beginning of the moving averages of the contrasted conditions. Accordingly, more general influences like an overall acceleration of RTs should influence the trials of both conditions equally, thereby leaving the interpretation of the difference between these conditions unaffected. Furthermore, random influences in the sequence of trials for a particular participant (e.g. if by chance a condition was presented six trials in a row) should be smoothed out by the calculation of an average effect course across participants. Following this line of reasoning, the interpretation of the effect course analysis should be applicable even for a fixed or block-wise presentation of conditions as long as condition or block order is counterbalanced across participants.

However, regarding the interpretation of the obtained clusters, a limitation of the chosen cluster-based testing approach for the effect course analysis needs to be considered. Cluster-based permutation tests cannot provide statistical certainty about the onset and respective offset of an effect^43,44^. They only provide statistical claims over the cluster size (cluster T-value), i.e. whether it is likely to obtain such a large or larger cluster, if the conditions would have been randomly assigned, i.e. by chance. The numerical onset/offset of a cluster is somewhat arbitrary, because it is determined by the threshold setting for the t-tests at the sample level. Choosing a more conservative alpha level for these t-tests compared to the chosen α = 0.1 would have resulted in narrower, choosing a more liberal alpha level in broader cluster. Concerning the identification of cluster onsets and offsets, further studies could therefore test whether existing methods in the field of neurophysiological data analysis can be extended to the effect course approach^51–53^.

Taken together, regarding the first goal of the present study, the proposed effect course analysis could recover previously described effect changes with practice, thereby demonstrating that it succeeds in unraveling qualitatively different effect courses. While this approach of validation was grounded in a descriptive comparison with previous work^15,46^, it however justifies the approach more in terms of a face-validity. Hence, to provide another benchmark, we also compared the effect course analysis with an already existing approach for the analysis of time courses of experimental effects, the segmentation of the experiment into different blocks and an inclusion of the block number as additional factor into statistical analysis, see Supplementary Material J. For this block analysis, the results strongly depended on the number of blocks the data was divided into, suggesting a critical influence of the somehow arbitrary process of choosing an appropriate block number, which determines the number of trials per block. In contrast, the effect course analysis, which further allows a continuous inspection of the time course of an effect in contrast to the block analysis, showed a remarkably larger robustness across different chosen window sizes. Nevertheless, it seems obvious that the choice of appropriate window sizes, sample thresholds and required sample sizes for the proposed effect course analysis needs to be addressed by further (methodological) studies.

Grounded on this validation on existing data, and regarding the second goal of the present study, the demonstration of a plasticity of task preparation processes, we applied the effect course analysis on our dataset, Study 2. Study 2 investigated how only cued, but not executed task sets (task cue-only) influence subsequent masked priming, if execution of cued task sets is postponed and these task sets therefore are required to be kept activated to a certain degree. The modulation of priming served as index how task sets were activated following task cue presentation^24^ and should therefore reflect how task cue processing as instance of task preparation changes during the experiment. The results showed semantic RT priming to be larger following a task cue associated with a semantic compared to a perceptual task set, but only at the beginning of the experiment, see Fig. 3.

To resume our hypotheses, we could think of two different effects of task cue-onlies on priming. First, participants have to keep cued task sets activated during the LDT, in order to be able to perform a possible induction task following the LDT. Accordingly, cued task sets sensitize priming and semantic priming should be larger following a task cue associated with the semantic compared to the perceptual task set. Second, and somehow related to the first hypothesis, activated task sets are in conflict with the required lexical task set of the LDT, and are therefore suppressed to resolve this conflict. The observed modulation partly supported the first hypothesis; priming was larger following a task cue associated with the semantic compared to the perceptual task set. However, this modulation rapidly vanished and could not be observed anymore after 15–20 trials (as these trial numbers reflect condition-wise, ordered trials, they should be more interpreted in a relative way, i.e. in terms of the modulation lacking after one-fifth/a quarter of the experiments’ total duration). Task cue processing therefore appeared to be plastically modified during the experiment (note that a traditional ANOVA on mean RTs/ERs did not reveal a significant modulation, see Supplementary Material A). The complete lack of the priming modulation later during the experiment cannot be readily explained by an increased conflict with the lexical task set of the LDT, which should have resulted in larger priming for perceptual than semantic task sets (due to a suppression of task sets to resolve the conflict)^16,17^. Hence, a qualitative change in task cue processing appears a more plausible explanation for this result pattern. Probably, at the beginning of the experiment, when induction tasks were not intensively practiced, participants activated task sets in response to task cues. Prepared task sets were kept activated during the LDT to facilitate later induction task performance, thereby modulating masked priming. Later, when task sets supposedly could be easily activated and implemented after sufficient practice, participants moved onto only remembering the task (cue) identity, and postponed task set activation until the need of task set execution was evident (i.e. when an induction task was presented). As preparing distinct task sets in response to task cues appeared to be no longer required to achieve a decent performance in induction tasks, only memorizing the task (cue) identity without preparing distinct task sets probably was beneficial in terms of reducing interference with the lexical task set of the LDT (with this regard, note that performance in the LDT started to improve only after the modulation of priming by cued task sets already lacked, see Supplementary Material H).

In line with this consideration, it might also be insightful to investigate the priming effect course separately per task set, as Fig. 3 only depicts the difference of both task set conditions. Regarding such a separate inspection, RT priming was maximal following a task cue associated with a semantic task set at the beginning of the experiment, and remained stable on a comparable level throughout the rest of the experiment. In contrast, RT priming for perceptual task sets was reduced and not included in a significant cluster at the beginning of the experiment, but started to increase after a couple of trials, resulting in comparable effect sizes compared to the semantic task set after around a third of the experiment (see Supplementary Material E). Hence, according to the attentional sensitization model of unconscious cognition^24^, at the beginning of the experiment, semantic priming was de-sensitized by perceptual task sets (and slightly boosted by semantic task sets). This modulation vanished after practice according to the hypothesized associated processing change, resulting in a similar level of priming for both task sets, implying the absence of a modulation of priming by attentional mechanisms.

Nevertheless, the vanishing of task cue effects on priming could also be explained in a different way. In line with theories assuming a shielding function of task sets^54–56^, it could be that with practice, task processing is more efficiently shielded from irrelevant information, thereby also reducing the influence of cued task sets on priming (regarding such a possible shielding mechanism, note that task set switch costs in induction tasks also vanished rapidly, see Supplementary Material I). This alternative explanation however requires the assumption that such a shielding mechanism specifically affects the modulatory influence of cued task sets on the masked semantic priming in the LDT, as overall priming was stable throughout the experiment (see Supplementary Material B). That is, the processing of the LDT target is not shielded generally from other influences, as the effect of masked primes on RT in the LDT did not change during the course of the experiment. Instead, shielding through increasing practice must specifically dampen the attentional sensitization of priming by task sets with progressing experimental duration. While it is difficult to theoretically decide between these two alternative explanations, a qualitative change in task cue processing with practice appears to be the more parsimonious account, as it does not require additional assumptions.

In summary, our results indicated that participants changed processing of task cues plastically during the course of the experiment, as practice resulted in an overall more efficient processing mode. Separate analyses depending on the performance in the practice block (see Fig. 4) moreover indicated that the time point when such postulated processing changes unfold their influence depended on the level of practice prior to the start of the experiment. If the practice performance was above-average, the modulation of priming appeared to be stronger, but also vanished more rapidly. In contrast, in the group with below-average practice performance, this modulation appeared to be somehow less stable at the beginning of the experiment, but more sustainable, indicating that the processes, which were discussed to cause the vanishing of this modulation, took longer to unfold their influence. Therefore, the plasticity of experimental effects might also depend on individual properties of participants, rendering the level of task practice upon start of an experiment an additional crucial factor for the investigation of practice effects. Note that such processing changes may not be required to be an active, strategic process, but might occur implicitly during the experiment, similar to the concept of automatization for performance improvements with practice^57^. Studying the course of an effect therefore appears to be a compelling method to shed light on how cognitive processing, in terms of a difference between experimental conditions^58^, is continuously adapted throughout an experimental session. The present study showed that at least concerning task preparation processes induced by task cues, such effect changes are relevant to consider. Upcoming studies could investigate how the importance of effect plasticity^2^ extends to other fields of cognitive psychology. Even if the time course is not in the focus of theoretical interest, effect course analyses are useful tools to get information about the temporal stability of an effect across the entire experiment. Finally, such inspections of effect courses are also of particular interest for the planning of experimental studies^15^. If effects are short-lived and vanish with continuing practice, it appears advantageous to design experiments with only a limited number of trials. In contrast, if effects only emerge at the end of a session, experiments could be designed to include a large number of trials or to administer a long practice session before the main experiment. However, note that for the detection of a processing change like in the current study, it is necessary to administer a sufficient number of trials to detect such a change. For instance, if we would have included only about a third of the trials in Study 2, we would have detected a modulation of priming by cued task sets, but not the theoretically relevant change of this effect, i.e. its plasticity.

To conclude, the present study applied a new model-free, non-parametric effect course analysis to study the plasticity of task cue effects on priming. This type of analysis was inspired by the analysis of neurophysiological and neuro-imaging data and included the application of cluster-based permutation tests, to establish at which time point during an experiment two experimental conditions differ. The effect course analysis, which was validated on previously reported effect changes with practice, showed task cue effects to change during the course of an experiment. Only at the beginning of the experiment, cued task sets modulated priming, indicating task sets to remain activated to a certain degree during the LDT, when task set execution was required later. However, this result pattern changed rapidly with the priming modulation lacking later during the experiment. Supposedly, after sufficient practice, participants presumably moved onto only remembering the task (cue) identity, and did not activate task sets in response to task cues, resulting in a missing priming modulation by task cues. This indicated that even the nature of task cue processing might be adjusted throughout an experiment. In line with these observed processing adaptations, we believe that examining the time course of an effect can serve as a useful tool to investigate how participants plastically adapt processing when they perform cognitive tasks. Even if the time course is not in the focus of theoretical interest, effect course analyses provide important information about the temporal stability of an effect across the entire experiment and are thus useful for designing new experiments.

## Method

### Study 1: re-analysis of data from Adelman et al. (2014)

To validate the proposed effect course analysis, we re-analyzed data from a large-scale priming study^46^, for which effect changes with practice were already demonstrated^15^. This study investigated masked form priming among different prime-target relations in a LDT. It includes data of 1015 participants, which all performed 840 trials. As the focus of the present study does not rest on the mechanisms of masked form priming, we will forego a detailed description of this study’s purpose and will only shortly describe the procedure of the masked LDT (for more detailed information please refer to the original article^46^). At the beginning of each trial, a fixation cross was presented for 300 ms, followed by a blank screen for 200 ms. Afterwards, a hash mask was presented for 500 ms, followed by the prime word in lowercase letters for 50 ms. After the prime, the target was shown in uppercase letters, until the participants responded or a response deadline of 2000 ms was crossed.

We chose this particular dataset among the other studies re-analyzed in Miller^15^, as for this study an initially increasing, peaking and further decreasing effect (word/nonword effect) and an initially absent, but further increasing effect (response repetition effect) were described. These two effects should allow testing whether the effect course analysis can recover changes in the slope of an effects increase (word/nonword effect) and that an effect is only observable after some trials practice (response repetition effect).

#### Pre-processing of data

We included the data of all 1015 available participants in the dataset. For the analysis of RT data, we excluded trials with an incorrect response as well as trials with a RT exceeding +− 2 SD of the individuals mean RT^48,59^. For the analysis of ERs, only trials with an RT outlier were excluded. Furthermore, for the analysis of the response repetition effect we also excluded the first trial of each participant as well as all trials with an error in the preceding trial. This resulted in an average number of 765.47 (SD = 47.2) and 780.64 (SD = 21.0) trials for the analysis of the word/nonword effect, respectively for RT and ER data. For the analysis of the response repetition effect, there were on average 704.65 (SD = 78.5) trials available for RT data and 715.83 (SD = 54.1) trials for ER data.

### Study 2

Study 2, including the effect course analysis, was pre-registered (https://osf.io/vek7a). Besides the investigation of the time course of task cue effects, we also performed a traditional ANOVA on averaged data as well as an analysis of single-trial data with linear mixed models^60^ and drift–diffusion models^8,9,61^ as pre-registered. These analyses, which did not account for practice changes within the experimental session and could not reveal a reliable modulation of priming by cued task sets, are reported in Supplementary Material A. Furthermore, EEG data was recorded in Study 2 to assess masked priming also on an electrophysiological level indexed by the N400 component^25^. However, as we did not observe a reliable modulation of N400 priming by task sets and to shorten manuscript length, pre-processing and analysis of EEG data are reported in Supplementary Material F. The local ethics committee of Ulm University approved Study 2 of the present study. The procedures used in this study are in compliance to the tenets of the Declaration of Helsinki. Informed consent was obtained from all individual participants included in the study.

#### Participants

A total number of N = 78 participants were recruited for Study 2. N = 11 participants were excluded for the following reasons: mean RT +− 2 SD of the sample mean in induction tasks or the LDT, n = 4; above-chance performance in a masked prime identification task, n = 6; 36% errors in induction tasks, n = 1. Note that the last exclusion criterion was not pre-registered, as we did not expect participants to perform that bad (participants intensively practiced all tasks before the experiment started). However, such a large error rate indicates deficits in performing the task (mean ER of all other participants was about 4%), justifying the exclusion of this participant. Accordingly, the final sample included N = 67 participants. Mean age was 22.99 (SD = 4.5) and n = 51 participants were female (76.1%).

#### Design and material

**Figure 5:**
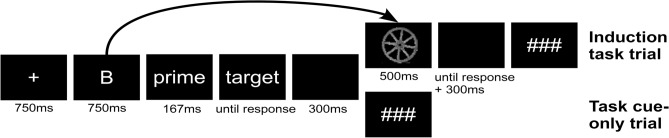
Experimental sequence in Study 2. Each trial started with the presentation of a fixation cross and a task cue (the letter “B” represents one of the two presented task cues in this experiment, “R” and “B”), followed by a lexical decision task (LDT). The LDT consisted of a prime interval (forward mask 100 ms, prime 33.5 ms, backward mask 33.5 ms) and the LDT target (represented by the placeholders “prime” and “target”, respectively). The induction task was presented after the LDT and cued task sets were therefore required to be kept activated to a certain degree during the LDT (represented by the arrow). Therefore, induction task trials (upper row) and task cue-only trials (lower row) did not differ until the end of the LDT and were collapsed for statistical analysis.

Study 2 incorporated two trial types, induction task trials and task cue-only trials, which were presented randomly and intermixed. Figure 5 shows a schematic overview of both trial types. At the beginning of each trial type, a fixation cross was presented for 750 ms. Afterwards, a task cue followed (750 ms), which cued either the semantic or the perceptual induction task. The task in the perceptual induction task was to classify a picture as depicting a round vs. elongated object, while the task in the semantic induction task was to classify a picture as depicting a living vs. non-living object. Task cues to indicate the perceptual and the semantic task were the letters R or B, respectively (counter-balanced across participants). As task cue conditions did not influence the pattern of masked priming (see Supplementary Material A), statistical analyses were collapsed across cue type conditions as in earlier work^17^. In task cue-only trials, following the task cue, the masked primed LDT was presented. The LDT consisted of a forward mask (100 ms, a string of 10 random uppercase letters), the prime word (33.5 ms) and a backward mask (33.5 ms, string of 10 random uppercase letters). Afterwards, the LDT target appeared, which stayed on the screen until a response was given. After the response to the LDT, a blank screen was presented for 300 ms. The LDT target had to be classified as word or as pronounceable, but meaningless nonword. Primes were always words and given a word as LDT target, primes could be either semantically related (e.g. table–chair) or unrelated with the target word (e.g. car–apple). In induction task trials the experimental procedure was the same compared to task cue-only trials until the end of the LDT. However, in induction task trials, after the LDT an induction task stimulus was presented for 500 ms on which the (in advance of the LDT) cued classification task had to be performed. After presentation of the induction task stimulus, a blank screen was presented until a response was given plus an additional 300 ms. At the end of each trial type, three hash marks were presented, and the next trial could be initiated with a self-paced button press. According to this experimental procedure, participants did not know until the end of the LDT, if they have to apply cued task sets. Cued task sets therefore were hypothesized to remain activated (to a certain degree) during the LDT. Due to the same sequence until the end of the LDT (where priming was assessed), both trial types were collapsed for statistical analysis.

Experimental conditions were equally balanced and Study 2 included a total number of 640 trials. The presentation of the different conditions (trial type x task set x prime condition) was completely randomized with no additional restrictions. N = 320 trials included a nonword as LDT target and were excluded from analysis. Among the n = 320 trials available for analysis, n = 160 trials were assigned to the semantic task set, and n = 160 trials to the perceptual task set (equally distributed across trial types, which however were collapsed for statistical analysis). Within each task set condition, there were equally many trials with semantically related and unrelated primes (each n = 80). Hence, for each task set condition the priming effect can be assessed at 80 trials. In a second step, for determining the manipulation of priming by cued task sets, this task set-wise priming effects can be further contrasted and the modulation of priming by task sets therefore bears on a maximum number of 80 condition-wise trials.

#### Practice block

To ensure that all participants had proper understanding of the experimental procedure upon the start of the main experiment of Study 2, they practiced the experimental procedure beforehand. In this practice block, participants received feedback about the correctness of their response. Participants first practiced the different tasks presented in the experiment separately, i.e. the LDT (8 trials), the perceptual (7 trials) as well as the semantic classification task (7 trials) in separate parts. Afterwards, they practiced the semantic and perceptual task together in one part (20 trials). At the end of the practice block, participants practiced the experimental procedure as it was presented in the main experiment (30 trials). If their performance in a part of the practice block indicated that participants were not yet familiar with the experimental procedure, they were asked to repeat this part until they achieved a decent performance indicating proper task understanding. While the experimenter therefore ensured that all participants achieved a decent performance at the end of the practice block, i.e. prior to the start of the main experiment, there were however inter-individual differences between participants’ performance in the practice block. Participants differed in how often they repeated parts of the practice block and the number of errors they made in the practice block. On average, participants performed 6.12 parts of the practice block (SD = 1.75, i.e. repeated about one part) and made 10.04 errors (representing around 8.4% of performed practice trials; SD = 7.87).

To investigate whether the observed modulation of priming by cued task sets in Study 2 depended on the level of task practice prior to the start of the experiment we splitted participants into two groups according to their performance in the practice block and calculated the effect course analysis separately for the two groups. To split participants into a group with “good” and “bad” practice performance, we calculated the average proportion of errors participants made per part of the practice block and performed a median split. Participants with an equal or higher proportion of errors than the sample’s median were considered to have a “bad” practice performance (n = 34), while the rest of participants were considered to have a “good” practice performance (n = 33).

#### Pre-processing of behavioral data

Trials with a nonword target served as filler trials and were excluded from analysis. Pre-processing of single trial data included the exclusion of RT outliers (exceeding +− 2 SD of an individual’s mean RT) as well as the exclusion of incorrect trials for the analysis of RT data. Available average trial numbers for RT analysis were 295.58 (SD = 7.6) and for ER analysis 306.66 (SD = 3.1).

### Effect course analysis

#### Summary of this type of analysis

**Figure 6:**
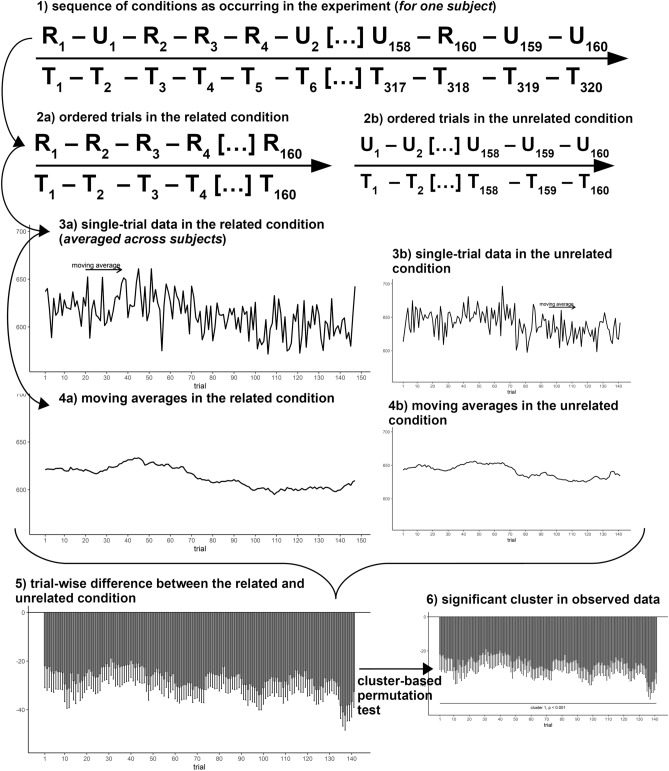
Schematic overview of the effect course analysis for the response time semantic priming effect in Study 2. Within the experimental session, related (represented by “R”) and unrelated (represented by “U”) prime-target conditions occur randomly, n = 160 trials for each condition, resulting in a total number of n = 320 trials (step 1, *T* represents trial indices). For this total number of trials, nonword targets were already excluded beforehand. The trials of both conditions are extracted and ordered according to their temporal occurrence, that is the next trial within a condition represents the next occurrence of this trial in the experiment (step 2a and 2b, *T* represents now ordered trial indices). Subsequently, the ordered single-trial data for related and unrelated prime-target pairs (step 3a and 3b) are smoothed by calculating moving averages (step 4a and 4b). Then, the two conditions are contrasted to depict the time course of the effect, i.e. the effect course of semantic priming. Note that the temporal dimension (trial) now only reflects the per-condition available trials. As not for all subjects data of all trials are available (outliers and incorrect responses were excluded), the trials numbers for this comparison are somehow smaller than n = 160 (step 5). These differences were thresholded at each sample/trial to build a cluster, where the semantic priming effect extends over time. The obtained cluster was compared against clusters in random permutations of the data to assess statistical significance (step 6). Note that while this figure depicts the effect course of priming in Study 2, this procedure can be generalized to every experimental effect contrasting two conditions.

The effect course analysis proposed in the present work was designed to reveal the development/time course of an experimental effect on a continuous time scale. Therefore, the effect is assessed at each step in the temporal dimension, i.e. single trials of the experimental conditions. As single-trial data is noisy, single-trial RTs/ERs were smoothed by calculating moving averages. Subsequently, at each time point, two experimental conditions were compared to assess the effect of this manipulation on RTs/ERs. This resulted in a representation of the time course of the investigated experimental effect depicted by the mean difference of both conditions at each time point/trial. In a second step, to assess how reliable the effect is over time, non-parametric cluster-based permutation testing (CBPT) was applied, which is a widely adopted method in the analysis of neurophysiological and neuroimaging data^43,45^. For our proposed effect course analysis of RT/ER data, the chosen approach for significance testing can be considered as a reduced variant of CBPT for neurophysiological data, where the data consists only of one dimension, i.e. time/trials and clusters are accordingly built according to temporal adjacency of reliable effects at the trial level. Reliability was assessed by thresholding the effects at the trial level, i.e. if the effect at a specific trial crossed a pre-defined threshold, it was considered significant. Subsequently, the obtained clusters in the observed data were compared against clusters observed in random permutations of the data and were considered significant, if it is unlikely to obtain such a (or a larger) cluster by chance. The following sections provide a more detailed description of the steps involved in this effect course analysis, but for more technical details, see also Supplementary Material G. Furthermore, all scripts required to conduct the effect course analysis implemented in *R*^62^ as well as the data were uploaded to the Open Science Framework (https://osf.io/6buz9/). Figures were created with the help of the package *ggplot2*^63^. A summary of the procedure is depicted in Fig. 6.

#### Calculation of smoothed response times/error rates

Smoothed RTs/ERs were calculated using moving averages. First, data was split according to the experimental factors (word/nonword, response repetition for the Adelman et al. (2014) dataset; and task set, semantic relatedness for our Study 2). In each cell of (all combinations of) the experimental factors, moving averages of RTs/ERs (sorted according to their temporal occurrence in the experiment) were calculated. That is, each vector of a particular cell of a particular subject contains the RTs/ERs ordered according to the temporal occurrence in the experiment (ordered from beginning to end) of this subject in this condition. Hence, for two trials to be included in a moving average, they are not required to be presented immediately after each other in the experiment (which would be unlikely if presented randomly), but the latter one needs to be the next occurrence of a trial of the particular condition. Subsequently, moving averages of these condition-wise, ordered trials were calculated. Therefore, a window size for the moving average was determined (see below) and slided across all trials. For example, if a chosen window size would have been seven, the moving average of a trial n would represent the average of trials {n−3, n−2, n−1, n, n + 1, n + 2, n + 3} of the corresponding experimental condition. This resulted in a representation of smoothed RTs/ERs (via calculation of moving averages) in that respective experimental condition. Hence, a “trial” for a moving average does not represent the index of that implemented condition among all trials of the experiment, but when this trial occurred relatively to the other implemented trials of that condition. If fewer trials were available as the given window size, for example for trials at the beginning/end of the particular condition, naturally only available trials were included in the moving average. For example, for the 2nd trial given a window size of seven, the moving average would consist of trials {1, 2, 3, 4, 5} of that condition. Hence, while no trial would be omitted, as only five trials are available, that moving average would naturally be calculated by averaging fewer trials (five) as e.g., in the middle of the condition-wise vector (which would be seven, i.e. the given window size of this example).

For Study 2, the maximum available number of trials in each condition (task set x semantic relatedness) was 80. To achieve an acceptable smoothing and to not conceal effect changes during the course of the experiment, we chose a window size of 1/5 of the condition-wise maximum available trial numbers. That is, the chosen window size was 80/5 + 1 = 17 for Study 2. One was added to the window size to achieve an odd number, which is required to obtain equally centered windows around a trial. For the re-analysis of word/nonword and response repetition effect in the Adelman et al. (2014) dataset, we chose the same window size, i.e. 17 trials. For this dataset, data of a very large number of participants was available, therefore reducing noise in across-subject averages. Accordingly, adequately smoothed RTs/ERs could be obtained with a more narrow window size compared to the maximum per-condition trial numbers.

#### Procedure for determining clusters in observed data

Subsequently, the moving averages were contrasted. As moving averages reflect the relative temporal occurrence of the trials of a particular condition, for this contrast to be reasonable the conditions are required to be balanced, i.e. each condition should include the same number of trials, which was the case for all analyzed effects in the present study. For the re-analysis of the Adelman et al. (2014) dataset, we contrasted nonword and word targets as well as response switches and response repetitions. The precise procedure of this contrasting procedure will be described for Study 2, as Study 2 involved testing the time course of an interaction effect. For Study 2, we first identified the priming effect separately for task sets. For determining this effect, the moving averages of related and unrelated trials were subtracted trial-by-trial wise. Note that due to pre-processing, there could be a different number of available trials and accordingly for the moving averages of both conditions. If this was the case, only trials were contrasted for which at least data for 80% of all subjects was available. The purpose of Study 2 was to determine the modulation of masked semantic priming by cued task sets and therefore rests on an interaction of the factors semantic relatedness × task set. Similar to further qualifying interaction effects in factorial designs^64^, interactions of factors were obtained by subtracting the condition differences. That is, the modulation of priming by task sets was obtained by first determining priming separately per task sets and then subtracting these task set-wise priming scores. To sum up, following effects/interactions were tested in the present study:

Re-analysis of Adelman et al. (2014):

1. *Word/nonword effect* = Nonword–word
2. *Response repetition effect* = Response switch–response repetition

Study 2:

1. *Modulation of priming by task sets* = (Related[Semantic]–Unrelated[Semantic])–(Related[Perceptual]–Unrelated[Perceptual]).

For each effect, clusters were formed. First, at the sample level (level of trials/moving averages), the data was thresholded. That is, for each moving average, two conditions were statistically compared using a dependent t-test. The dependent t-test was considered significant, if *p* < 0.1. Note that we chose a slightly more liberal threshold than the conventional α = 0.05 to account for variability in the data on the trial (or sample) level (especially for Study 2, for which a smaller sample size was available). However, note that the choice of the threshold on the sample level does not affect the validity of the CBPT procedure, as only the cluster T-value and not the single comparisons are subject to the second-level permutation tests^43,44^. If a test on the sample level exceeded this pre-defined threshold (i.e. *p* < 0.1), it was considered significant. Clusters were simply built by summing the t-values of all adjacent significant trials/moving averages. For example, if out of 15 trials the trials 2, 3, 4, 5, 8, 9, 10, 11, 12, 14, 15 would have been significant, three cluster would have been built: cluster 1: trials 2–5, cluster 2: trials 8–12, cluster 3: trials 14–15. After cluster forming, cluster T-values were obtained by summing the t-values of all trials included in the respective cluster.

#### Cluster-based permutation tests

Finally, the observed clusters were tested against a null hypothesis assuming an exchangeability of conditions, to assess how likely it is to observe a similar (or larger) cluster if conditions are randomly assigned^43–45^. Therefore, random permutations of the data were generated. In each random permutation, the levels of each condition were randomly shuffled to mimic a random assignment of conditions. That is, separately for each participant, all trials in level A and all trials in level B were randomly shuffled, i.e. could either keep their initial assignment or could flip assignments. In these random partitions, clusters were built as described above. Of all clusters in a permutation, the maximum cluster T-value of all positive clusters and the minimum cluster T-value of all negative clusters were extracted. This procedure was repeated for all permutations. After the calculation of cluster T-values in random permutations, the proportion of clusters of all permutations with a cluster T-value equally large or larger (for positive clusters, equally small/smaller for negative clusters) than a cluster obtained in the observed data was calculated, which represent the *p*-value of the respective observed clusters. In the present study, we used 5000 permutations for Study 2 and 1000 permutations for the re-analysis of the Adelman et al. (2014) dataset. We chose a smaller number of permutations for the Adelman et al. (2014) dataset, as according to the substantially larger sample size compared to Study 2, calculations of cluster-based permutation tests took quite long. However, all clusters in this dataset were far below the significance threshold (see the “Results” section), rendering it unlikely that the significance of these clusters is the consequence of a lowered precision due to a smaller number of permutations. Clusters were considered significant, if *p* < 0.05.

#### Plotting the effect course analysis

For the graphical depiction of the effect course analysis, we plotted the difference between the to-be-compared conditions at each trial (using bar plots). To display the statistical precision of this difference, associated standard errors are plotted alongside the bars as whiskers. Furthermore, standardized effect sizes of the difference^65^,mean(x_1_−x_2_)/SD(x_1_−x_2_), are displayed within these plots as solid lines. Comparing the raw differences and effect sizes should enable evaluating whether changes in an effect are associated with an increased/decreased variability during the experiment. For example, if an (raw) effect increases but this increase is associated with an increase in variability, the change of the standardized effect size would be smaller compared to the change in the raw difference. Therefore, both raw effects (difference between conditions) and standardized effect sizes are shown to be able to estimate the impact of such influences.

### Ethics declaration

The local ethics committee of Ulm University approved Study 2 of the present study. The procedures used in this study are in compliance to the tenets of the Declaration of Helsinki. Informed consent was obtained from all individual participants included in the study.

### Supplementary Information

Supplementary Information.

## Data Availability

The data and scripts to re-produce the effect course analysis as well as the other analyses reported in the present work were uploaded to the Open Science Framework and are publicly available at: https://osf.io/6buz9/.
