# Supplementary material for: Dynamics of task preparation processes revealed by effect course analysis on response times and error rates
Source: Sci Rep. 2024 Feb 21;14:4249. doi: 10.1038/s41598-024-54823-1 (PMC10879509; doi:10.1038/s41598-024-54823-1)
Supplement: Supplementary file 1 — Supplementary Information. [file 41598_2024_54823_MOESM1_ESM.pdf]

Supplementary Material

Dynamics of task preparation processes revealed by effect course analysis on response times and error rates

Alexander Berger[1*], Wilfried Kunde[2], and Markus Kiefer[1]

[1]Ulm University, Department of Psychiatry, Ulm, Germany

[2]University of Würzburg, Department of Psychology, Germany

A: Analysis of behavioral data in Study 2 ignoring practice-related changes 2

B: Figures for the “raw” priming effect course in Study 2 4

C: Figures for the effect course analysis of error rates in the re-analysis of the Adelman et al. (2014) study and Study 2 5

D: Re-analysis of the response repetition effect in the Adelman et al. (2014) dataset with a different pre-processing of RT data 7

E: Figures for the priming effect course in Study 2 separately per task set 10

F: N400 priming in Study 2.. 12

G: Technical description of the effect course analysis.. 15

H: Time course of overall LDT and induction task performance in Study 2 17

I: Effect course of task set switch effects in induction tasks in Study 2 20

J: Comparing the effect course analysis with a more traditional analysis approach separating the experiment into blocks 21

K: Re-analysis of the effect course analysis in Study 2 depending on previous trial type and task set sequence 24

Supplementary references 26

A: Analysis of behavioral data in Study 2 ignoring practice-related changes

Traditional ANOVA analysis

For conventional analysis of response time (RT) and error rate (ER) data in Study 2, we calculated mean RTs / ERs separately per condition, i.e. for the factors semantic relatedness x task set. Furthermore, to demonstrate that the cue type did not influence the pattern of the modulation of priming by task sets, we further included the between-subjects factor cue type in this analysis, i.e. how the task cues were assigned to the semantic and perceptual task set. Compatible task cues: task cue R – perceptual task set, "rund" decision; task cue B – semantic task set, "belebt" decision; incompatible task cues: reversed assignment, B – perceptual, R – semantic task set.

For RTs, there was only a significant effect of semantic relatedness, $F(1, 65) = 135.83$, $p < .001$. The effect of interest, the interaction of task set and semantic relatedness, did not reach significance, $F(1, 65) = 1.51$, $p = .224$. No other effect reached significance as well (all *p*s > .433). However, descriptively, there was a slight trend towards more RT priming following a semantic compared to a perceptual task set, see *figure S1*.

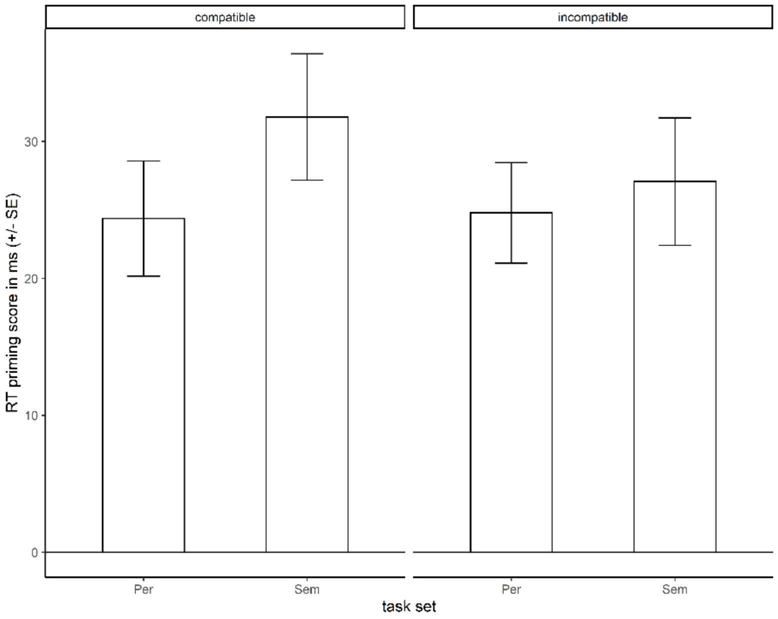

Figure S1 Priming effect for response times in Study 2, separately per task set and cue type conditions.

For the analysis of ERs, there was a significant priming effect, $F(1, 65) = 79.06$, $p < .001$, but no other effect reached significance (all *p*s > 0.065), including the theoretically relevant modulation of priming by task sets ($p = .277$).

Linear mixed models

We also performed linear mixed model (LMM) regression analysis using *R* packages *lme4* and *lmerTest* (Bates et al., 2014; Kuznetsova et al., 2017). The factors were contrast-coded:

Cue type: compatible = 0.5; incompatible = -0.5; task set: semantic = 0.5; perceptual = -0.5; semantic relatedness: related = 0.5; unrelated = -0.5.

All factors were included as fixed factors in the model. Furthermore, we included subjects as random intercepts. For the analysis of RTs, task set and semantic relatedness were also included as random slopes. For the analysis of ERs, inclusion of random slopes yielded to failed model convergence and the random effects structure for the analysis of ERs therefore only included random intercepts. ERs were estimated using a generalized linear model (GLMM) with a binomial link function.

For the analysis of RTs, the corresponding LMM only revealed a significant intercept and main effect of semantic relatedness, but the crucial interaction task set x semantic relatedness did not reach significance (β = -4.87, t = -1.20, p = 0.230), as well as all other interactions and main effects (all other ps > 0.428). For ERs, only significant effects for the intercept and semantic relatedness were observed as well. The interaction task set x semantic relatedness was not significant (β = -0.20, z = -1.20, p = 0.229), as well as all other effects (all ps > 0.091).

Drift-diffusion models

We also analyzed the modulation of priming by cued task sets on drift rates *v* and non-decision times *t0* using drift-diffusion modelling (Ratcliff & McKoon, 2008; Voss et al., 2013). In our previous study, the modulation of priming by task sets was confined to drift rates (Berger et al., 2022). We estimated drift rates and non-decision times varying for task set and semantic relatedness (the decision threshold *a* was fixed) with Bayesian hierarchical drift-diffusion models (HDDM; Wiecki et al., 2013) and extracted estimated model parameters (drift rates, non-decision times), which were subsequently handed to a Bayesian repeated-measure ANOVA (JASP Team, 2020; Wagenmakers et al., 2017). The Bayesian ANOVAs included the within-subject factors semantic relatedness and task set as well as the between-subject factor cue type.

Bayesian ANOVAs revealed extreme evidence for a main effect of semantic relatedness on drift rates (BF = $6.62*e^{27}$), but no evidence for any other effect (all BFs < 1.07), including the theoretically relevant interaction task set x semantic relatedness (BF = 0.62). For the analysis of non-decision times, there was moderate evidence for a main effect of semantic relatedness (BF = 5.49), but no evidence for any other effect (all BFs < 1.02, including the interaction task set x semantic relatedness, BF = 0.19).

B: Figures for the “raw” priming effect course in Study 2

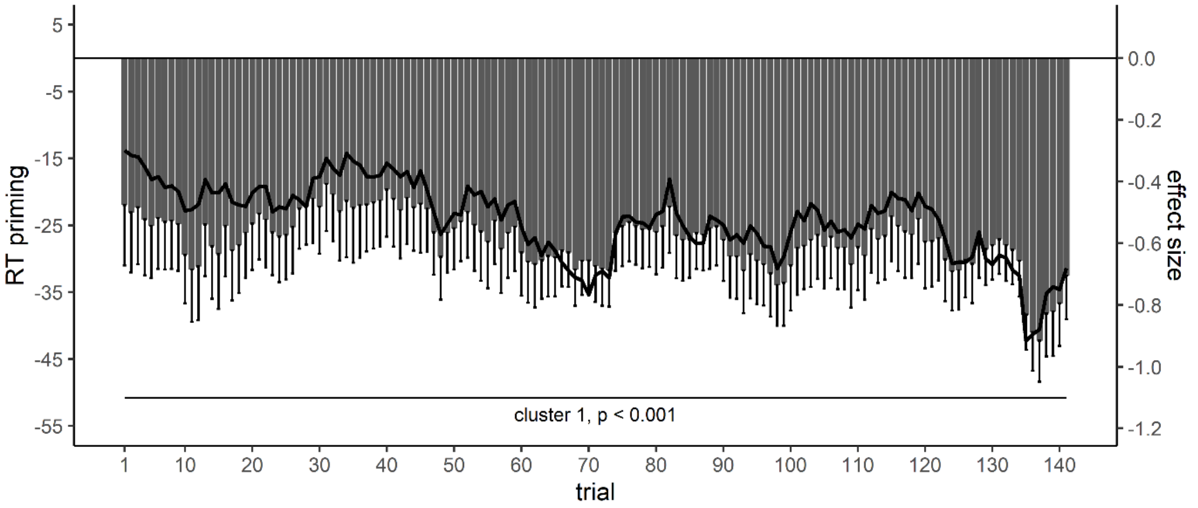

Figure S2 Effect course of priming for RTs in Study 2. The bars show the difference of related minus unrelated trials, the solid line the effect size of this difference. Cluster $T = -592.58$, $p < .001$, trials 1 – 141.

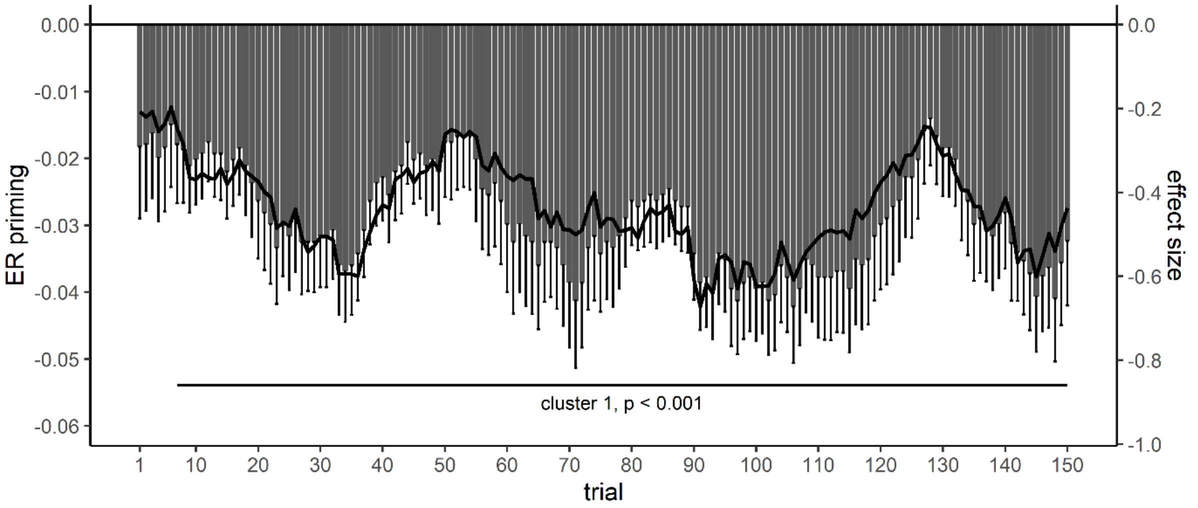

Figure S3 Effect course of priming for ERs in Study 2. Cluster $T = -522.45$, $p < .001$, trials 7 – 150.

C: Figures for the effect course analysis of error rates in the re-analysis of the Adelman et al. (2014) study and Study 2

Adelman et al. (2014)

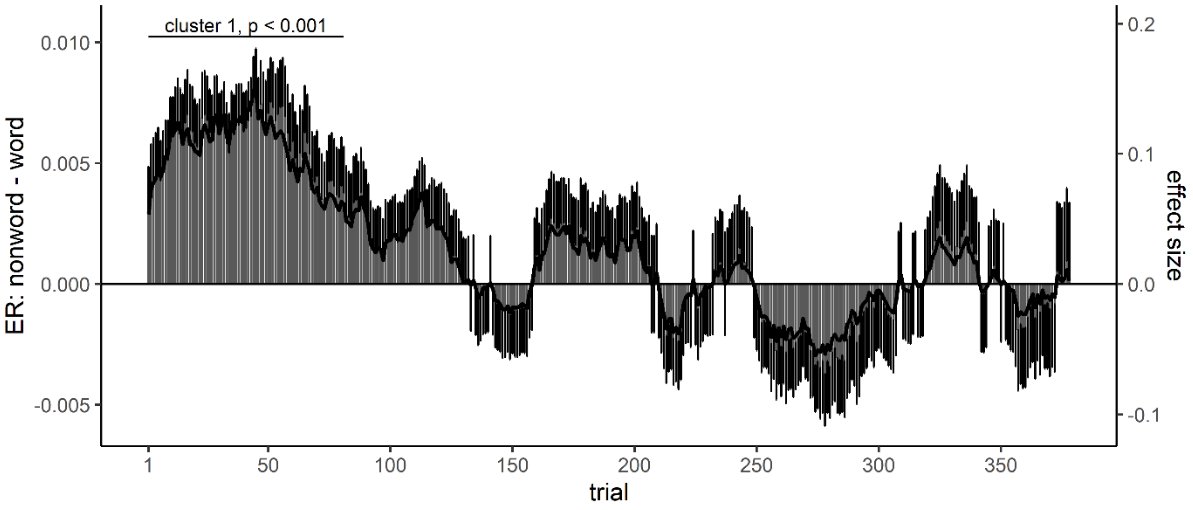

Figure S4 Effect course analysis for ERs for the word / nonword effect in the Adelman et al. (2014) study. Cluster $T = 264.85$, $p < .001$, trials 1 – 81.

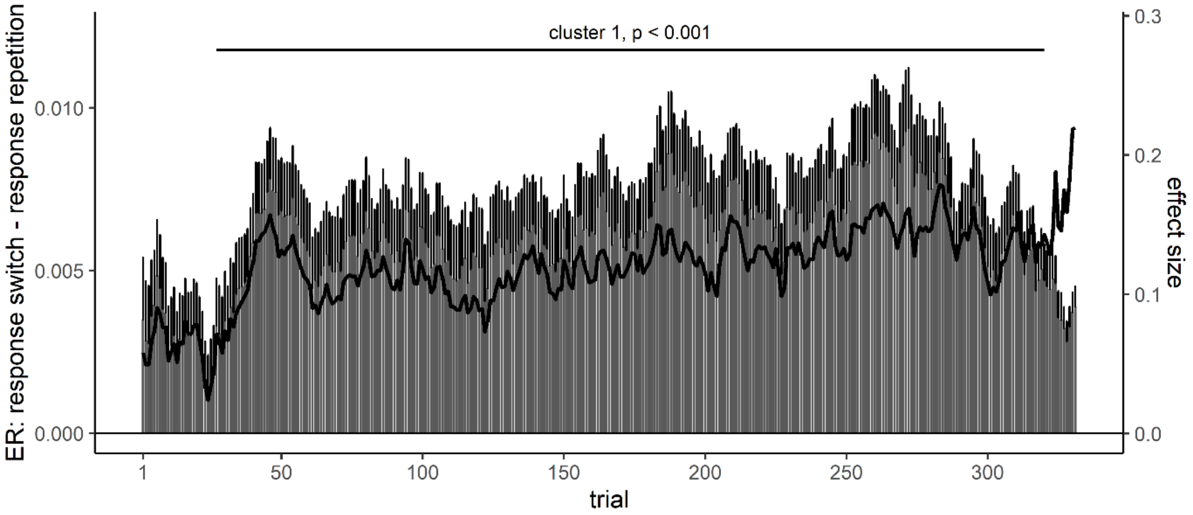

Figure S5 Effect course analysis for ERs for the response repetition effect in the Adelman et al. (2014) study. Cluster $T = 1120.91$, $p < .001$, trials 27 – 320.

Study 2

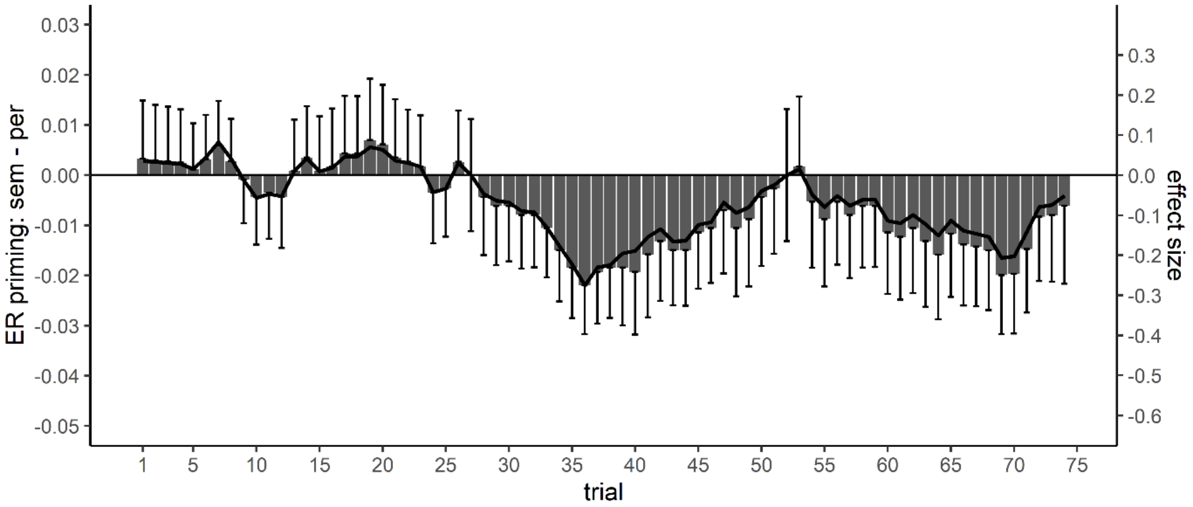

Figure S6 Effect course analysis for ERs for the modulation of priming by task sets in Study 2. Negative values indicate larger semantic priming following task cues associated with a semantic compared to a perceptual task set. No significant cluster was observed.

D: Re-analysis of the response repetition effect in the Adelman et al. (2014) dataset with a different pre-processing of RT data

For the re-analysis of the response repetition effect in Adelman et al. (2014), we observed a slightly different time course compared to the one described by Miller (2023). Miller described the effect to be absent for around the first 20-30 trials, while we observed for these initial trials an effect of around 5ms (see *figure 2* in the main text). This appears to be the consequence of a different pre-processing of RT data between both analyses.

In the following, we will show that the same effect course can be obtained by only excluding incorrect responses (as well as, naturally, the first response of each participant). For this purpose, we will further display the effect course only beginning with the fifth trial, for which the first response repetition effect is displayed in Miller (2023). Furthermore, note that the focus does solely rest on the shape of the effect course and we therefore ran only 100 permutations. Accordingly, the number of permutations was too low to establish precision about the significance of clusters and cluster *p*-values should therefore not be considered relevant.

Figure S7 shows the effect course with the adapted data pre-processing. As one can see, like reported in Miller (2023), the response repetition effect is now missing for the initial trials.

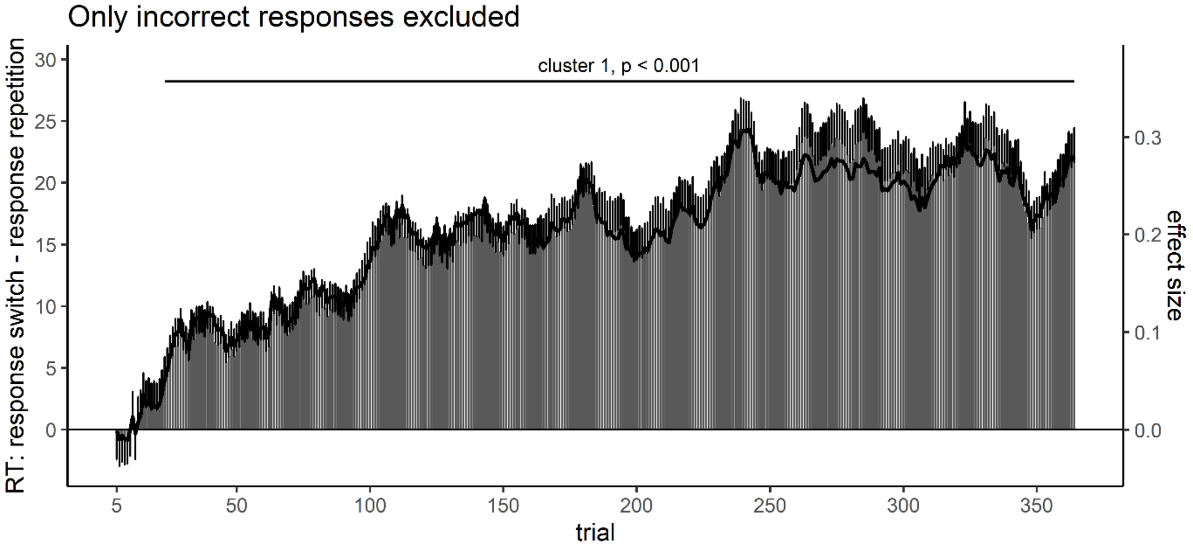

Figure S7 Effect course for the response repetition effect if only incorrect responses were excluded during data pre-processing. The effect is now missing for the first trials.

If one might wonder, why (raw) effects are still numerically larger compared to Miller (2023), e.g. Miller reported an effect of around 5ms after 100 trials, while in the present effect course analysis there is an effect of around 10ms at this time point; this is the consequence of calculating moving averages in the present analysis. When calculating *cumulative* moving averages, the initial lack of the effect is carried onto the effect sizes at later trials, therefore reducing these effects. For the sake of completeness, *figure S8* therefore shows the same analysis when cumulative moving averages are calculated instead, that is repeating the same analysis as in Miller (2023). As one can see, with the adopted pre-processing and the calculation of cumulative moving averages, the previously described effect course can be replicated. Note that the difference between raw and standardized effect sizes (solid line) in *figure S8* (in contrast to *figure S7*) is a consequence of calculating cumulative moving averages. Standardized effect

sizes profit more strongly from the inclusion of additional trials (due to a reduced variability), therefore the course of the standardized effect size increases more “steeper” throughout the experimental session (the standardized effect size is scaled at the maximum of the raw effect size).

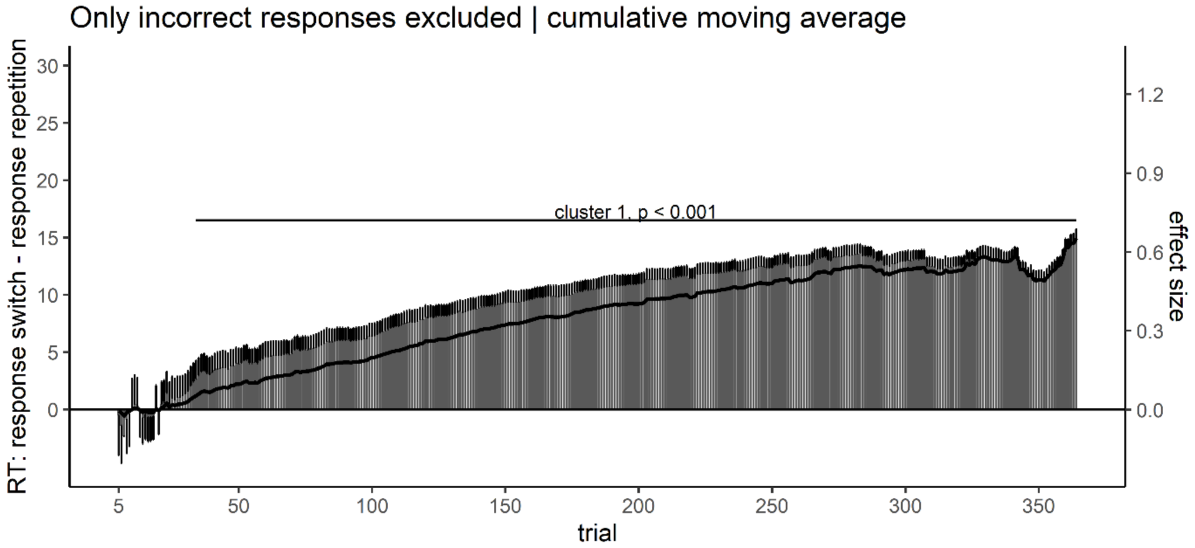

Figure S8 Effect course using cumulative moving averages for the response repetition effect if only incorrect responses were excluded during data pre-processing.

The previous analyses demonstrated a slightly different (shape of the) effect course with a different pre-processing of the data, i.e. whether RT outliers are retained (the present supplementary analysis) or excluded (the analysis reported in the main text) for the effect course analysis. However, such pre-processing influences can also be quite large, indicating that an appropriate pre-processing of data is indeed necessary before performing an effect course analysis. Regarding this aspect, see *figure S9*, which shows an effect course analysis of the response repetition effect, when no trials were excluded (besides the first trial, where no response switch / repetition naturally could occur), i.e. where both correct and incorrect responses were included (as well as RT outliers). In this analysis, the response repetition effect lacked at all. This disappearance of the effect is not surprising, as it can be expected that different processes lead to a correct versus an incorrect response and it therefore is likely that for these two processes, the influence of the response given in the previous trial differs as well. Accordingly, due to the same rationale as it is usual assumed in the traditional analysis of RTs / ERs (cf. Berger & Kiefer, 2023; Cousineau & Chartier, 2010; Kahveci et al., 2023), we suggest to exclude trials before performing an effect course analysis, for which it cannot be assumed that cognitive processes took place regularly. To ensure that the effect course of the cognitive process *of interest* is investigated; an exclusion of trials, which may contaminate the interpretation of this effects (like e.g. outliers or incorrect responses for the analysis of RTs) therefore seems to be crucial.

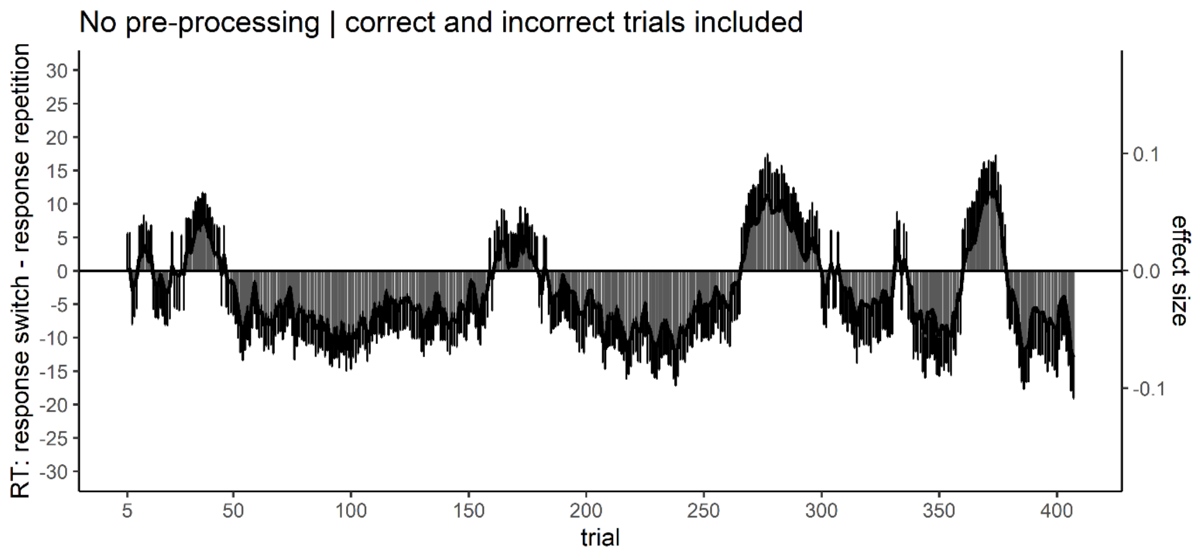

Figure S9 Effect course for the response repetition effect if both correct and incorrect responses were included, i.e. without additional pre-processing of data. When incorrect trials were not excluded, the response repetition effect lacked at all.

E: Figures for the priming effect course in Study 2 separately per task set

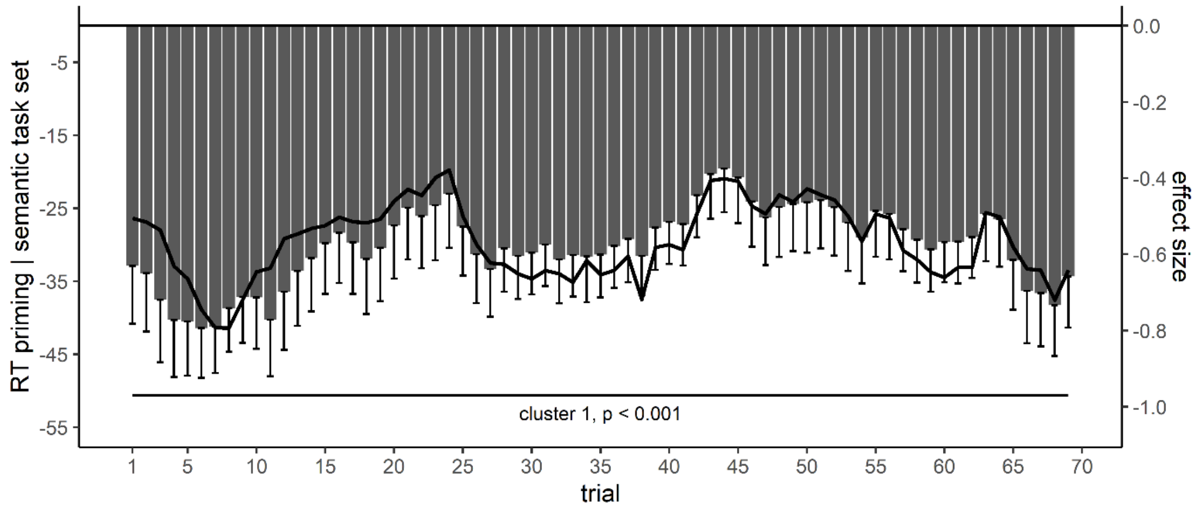

Figure S10 Effect course of priming for RTs in Study 2 following task cues associated with a semantic task set. Cluster T = -315.13, p < .001, trials 1 – 69.

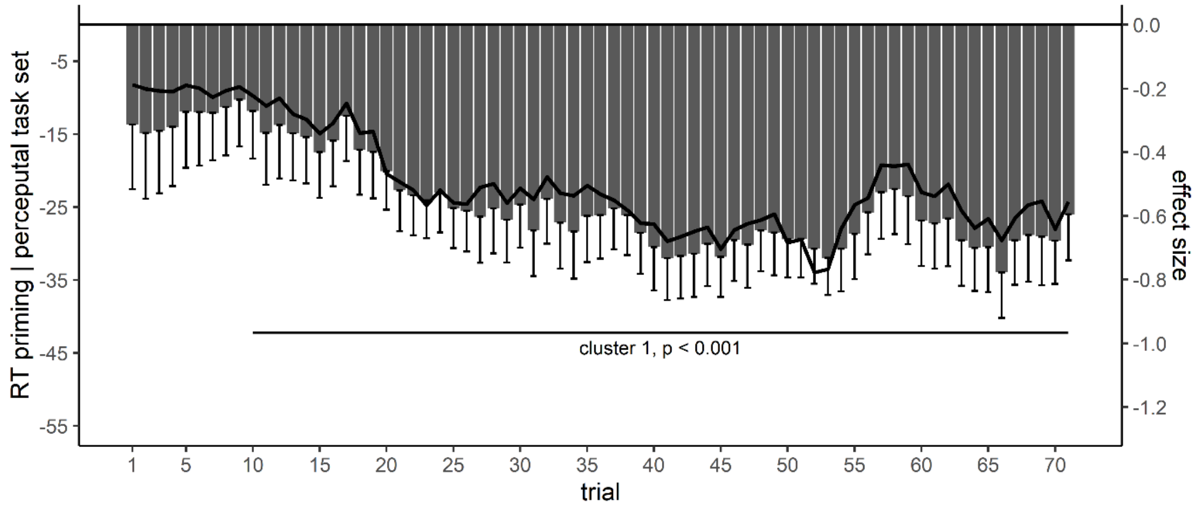

Figure S11 Effect course of priming for RTs in Study 2 following task cues associated with a perceptual task set. Cluster T = -267.79, p < .001, trials 10 – 71.

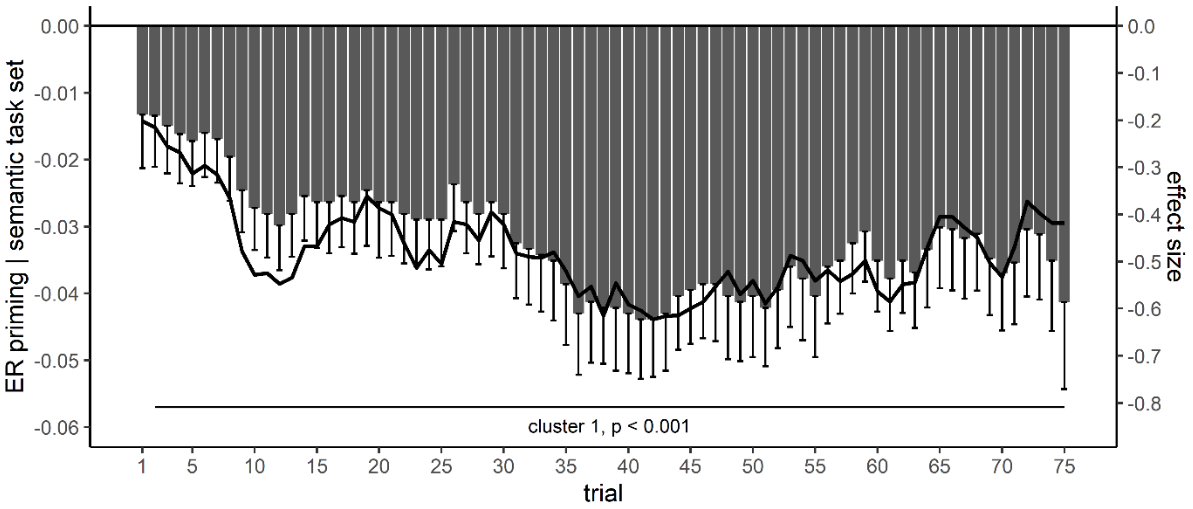

Figure S12 Effect course of priming for ERs in Study 2 following task cues associated with a semantic task set. Cluster $T = -289.50$, $p < .001$, trials 2 – 75.

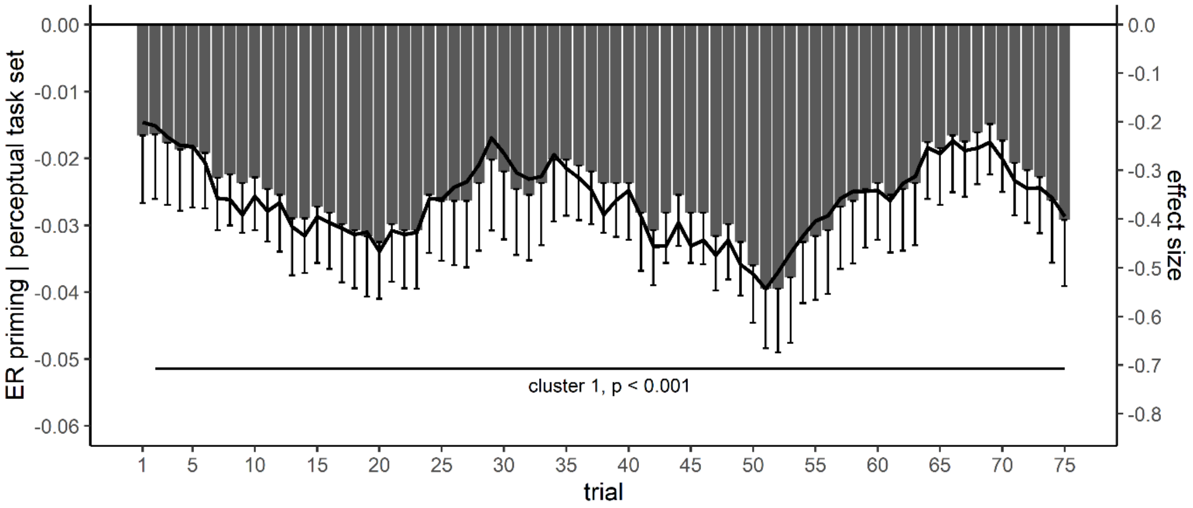

Figure S13 Effect course of priming for ERs in Study 2 following task cues associated with a perceptual task set. Cluster $T = -218.37$, $p < .001$, trials 2 – 75.

F: N400 priming in Study 2

In Study 2, electrophysiological (EEG) data was recorded to assess how task cues and associated task sets modulate masked semantic priming on an electrophysiological level. For investigating masked priming in EEG data, we extracted the N400 event-related (ERP) component (Kiefer, 2002) and tested, how the magnitude of N400 priming is influenced by (cued) task sets similar to previous work (Berger et al., 2022; Kiefer & Martens, 2010; Martens et al., 2011).

Recording and pre-processing of EEG data

We recorded EEG from 64 equidistant, sintered Ag/AgCl electrodes. Recording was performed using a BrainAmp amplifier and BrainVision Recorder software (BrainProducts, Gilching, Germany) with a sampling rate of 500 Hz. Reference and ground electrode were placed between FCz and Cz as well as AFz and Fz, respectively. Impedances were held below 5 kΩ.

Pre-processing of EEG data was performed using Fieldtrip software, version 20201214 (Oostenveld et al., 2011). First, continuous EEG data was low-pass (30 Hz), high-pass (0.1 Hz) and DFT filtered (50 Hz, 100 Hz, 150 Hz). Subsequently, EEG data was segmented in a time interval of -1.417 sec to +1 sec relative to lexical decision task (LDT) target onset, i.e. ranged from 250ms after onset of the fixation cross until 1000ms after target onset. Overlapping trials were excluded. Afterwards, channels with a bad signal were identified by visual inspection and replaced by the mean of all surrounding channels. PCA correction was applied to remove ocular artifacts. Segmented data was re-referenced to an average reference and baseline corrected for a baseline window starting 300ms after fixation cross onset and ending 50ms before fixation cross offset. Accordingly, the baseline window should not include any task-related activity. Finally, an automatic artifact rejection was performed using a z-value of 30.

Statistical analysis of EEG data

Before statistical analysis, trials with an incorrect response as well as trials with an RT outlier were excluded, as they could represent a deficit in task processing (Berger & Kiefer, 2023). To identify which electrodes and time intervals reflected N400 priming in the present data set, we performed cluster-based permutation tests (CBPT; Maris & Oostenveld, 2007), contrasting trials with semantically related and unrelated prime-target pairs. Subject-wise averages of related and unrelated trials were compared on the sample level with two-sided dependent t-tests ($\alpha = 0.05$). The (electrode, time)-wise t-values were clustered according to FieldTrip's default cluster forming algorithm, using a neighborhood structure indicating each surrounding electrode as a neighbor of a particular electrode. To restrict clusters to local maxima, a significant t-test at a (electrode, time)-pair was only included in a cluster if at least the t-tests at two neighboring electrodes were significant as well. CBPT were performed in a time interval of -300ms to +1000ms relative to LDT target onset. We calculated 1000 random permutations and clusters were considered significant, if $p < .05$.

CBPT revealed six significant clusters, three showing more positive ERPs for related than unrelated prime-target pairs and three clusters with the reversed polarity. By visual inspection, we identified the first positive cluster to reflect N400 priming. The cluster extended from 426ms to 654ms relative to LDT target onset, peaking at 500ms at electrode CP3. It included 36 electrodes, mainly spanning over parietal, central and frontal electrodes. For a graphical depiction of this cluster, see *figure S14*.

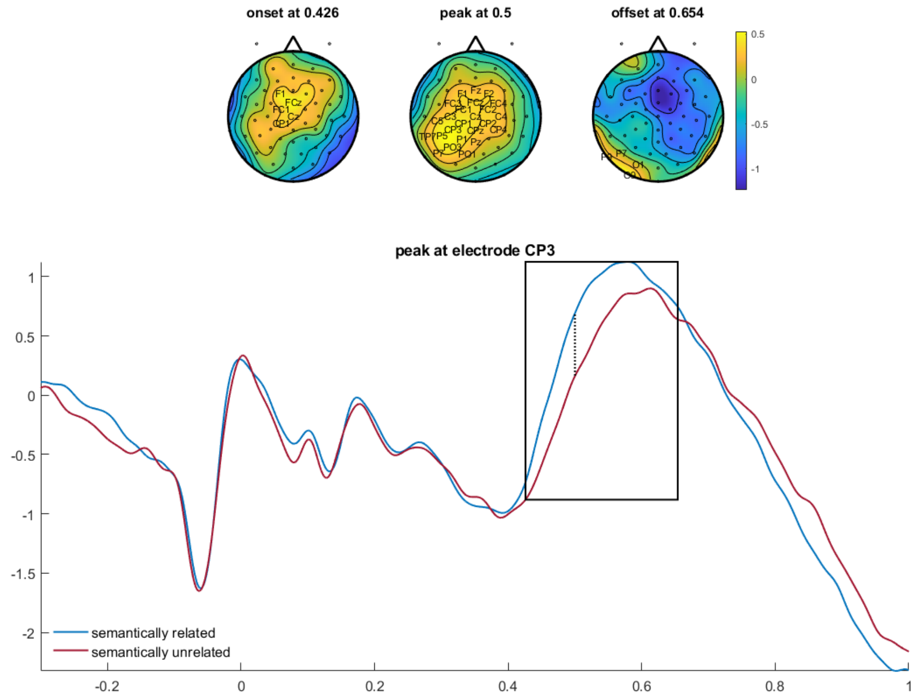

Figure S14 Significant cluster reflecting masked semantic priming in Study 2. x = 0 represent the LDT target onset. Topographical plots at the top represent the difference of related and unrelated trials at the onset, peak and offset of the cluster, respectively. At the bottom, the time course of grand-averaged ERPs at the peak electrode is shown. The box represents the cluster extents at the temporal level, while the dotted vertical line depicts the cluster's peak.

To statistically compare how N400 priming differed between (cued) task sets, we extracted subject-wise averages of ERPs in this cluster, i.e. averaging across the included time interval and electrodes, separately for the two task set and semantic relatedness conditions, as well as brain hemisphere[1]. Subsequently, these averages were handed to an ANOVA to test whether N400 priming differs for task set. The ANOVA included the within-subjects factors semantic relatedness, task set and brain hemisphere as well as the between-subjects factor cue type (compare *Supplementary Material A*).

No theoretically relevant interaction including task set and semantic relatedness reached significance (all $ps > .302$). Only the main effects of hemisphere, $F(1, 65) = 48.32$, $p < .001$, task set, $F(1, 65) = 8.94$, $p = .004$, semantic relatedness, $F(1, 65) = 21.60$, $p < .001$, as well as the interaction hemisphere x semantic relatedness, $F(1, 65) = 4.48$, $p = .038$, were significant. ERPs were more positive over the right hemisphere, for perceptual task sets and for related prime-target pairs. Furthermore, the N400 priming effect was more pronounced over the left hemisphere. *Figure S15* shows a graphical depiction for the modulation of priming by task sets, indicating that N400 priming was comparable for semantic and perceptual task sets.

[1] As pre-registered, we analyzed N400 priming including the factor brain hemisphere. Therefore, all electrodes included in the cluster over the left hemisphere were averaged and all electrodes over the right hemisphere were averaged.

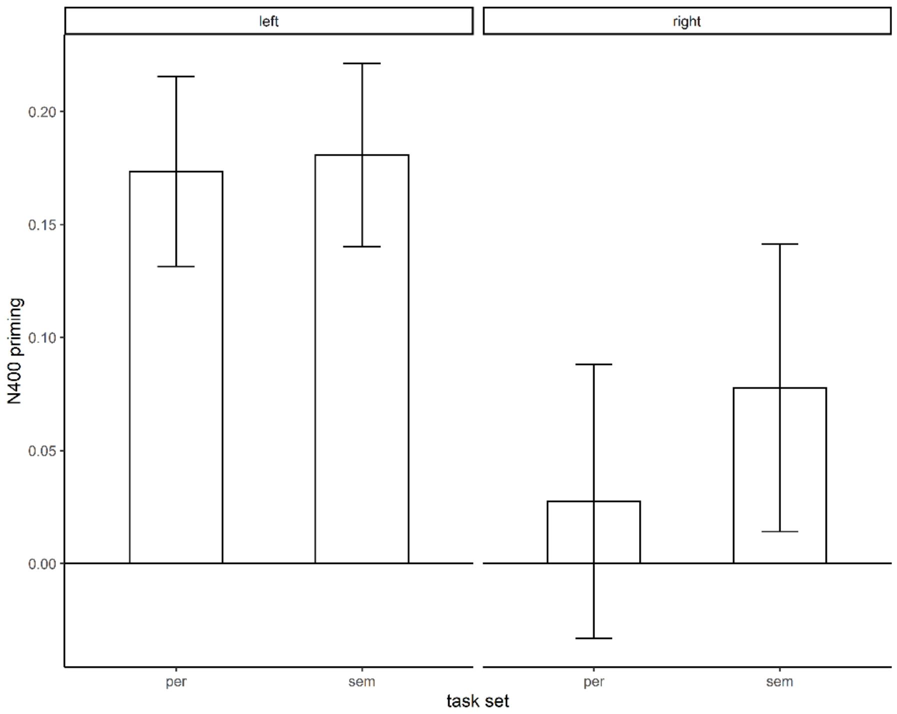

Figure S15 N400 priming (semantically related minus semantically unrelated) separately for task set and brain hemisphere.

G: Technical description of the effect course analysis

This section provides a more detailed, technical overview over the effect course analysis. For a more general overview, see the main text. Furthermore, all scripts and functions used for performing the effect course analysis were uploaded to the Open Science Framework (https://osf.io/6buz9/).

Following steps are performed in the effect course analysis to investigate the effect of one experimental factor:

1) Data is extracted for both conditions of the factor, separately for each subject and ordered according to their temporal occurrence (trial number). That is, a subsequent data point represents the next occurrence of this condition in the experiment. Separately for each subject and each condition, moving averages are calculated.
2) For calculation of moving averages, first a window size for the moving average is specified. The window size needs to be an odd number to achieve symmetric centering around one trial. Moving averages are calculated for all trials by the mean of all trials included in the particular moving average, that is for a trial n and an odd window size w ($w \geq 7$), the moving average is calculated as[2]:
   moving average_n = mean($\text{trial}_{n\text{-wh}}$; $\text{trial}_{n\text{-wh+1}}$; $\text{trial}_{n\text{-wh+2}}$; …; trial_n; …; $\text{trial}_{n\text{+wh-2}}$; $\text{trial}_{n\text{+wh-1}}$; $\text{trial}_{n\text{+wh}}$), with $wh = (w\text{-}1) / 2$
   For trials were not all trials for the given window size are available, e.g. at the beginning / end, only available trials are used, e.g. for trial n = 3 with a window size 9: moving average_3 = mean(trial_1; trial_2; trial_3; trial_4; trial_5; trial_6; trial_7)
3) In a next step, cluster-based permutation testing (CBPT) is performed. First, the observed data is thresholded, that is at each sample (trial / moving average), both conditions are statistically compared. For this purpose, a paired t-test is calculated which is considered significant if the p-value is smaller than a given threshold (here $\alpha = 0.1$). Only data, for which for at least 80% of participants data is available, is thresholded. The output is a list indicating for each sample, if it was significant or not. Second, clusters are formed in the observed data. Therefore, the t-values of (temporally) adjacent significant samples are summed.[3] That is, t-values of subsequent significant samples are summed, until the next sample is no longer significant, and that sequence of significant samples form a cluster. A cluster is represented by the cluster T-value, which is the sum of the t-values of all samples included in this cluster.
4) After the clusters in the observed data are determined, clusters are calculated in random permutations of the data. That is, separately for each subject, the two conditions of the experimental factor are randomly permutated. Consider all possible random permutations for an example of two subjects:

**Subject**	**Initial condition**	**Perm. 1**	**Perm. 2**	**Perm. 3**	**Perm. 4**
1	*A*	A	A	B	B
	B	B	B	A	A
2	*A*	A	B	A	B
	B	B	A	B	A

[2] For computational efficiency, the moving averages are in fact computed using a convolution.
[3] Naturally, temporally adjacent samples are only summed, if the difference of both conditions has the same sign, i.e. if the effect points in the same direction.

As for a larger number of subjects, there is a huge number of possible permutations (for N = 50 participants for example $2^{50} \approx 1.1*e^{15)}$, not all possible permutations are generated. A Monte-Carlo approach is chosen and only a pre-defined number of random permutations are drawn (Maris & Oostenveld, 2007). Usually, for EEG data, a minimum number of 800 permutations is considered sufficient (Pernet et al., 2015). In these random permutations of the data, clusters are built as described in step 3). Separately for positive and negative clusters, that is for clusters where condition A > condition B and for clusters were condition A < condition B, respectively, the maximum (/ minimum) cluster *T*-value is extracted. If there is no positive / negative cluster for a given permutation, the extracted *T*-value is set to 0. This results in two distributions of *T*-values in random permutations of the data, one for positive clusters and one for negative clusters.

5) Finally, the *p*-values of the observed clusters are calculated. Separately for positive and negative observed clusters, it is calculated how many extracted *T*-values in random permutations yield an equally large or larger (equally small or smaller for negative clusters) *T*-value. This number is divided by the number of permutations, which yields the *p*-value of that cluster. That is, for two observed clusters, one positive cluster C_p and one negative cluster C_n, and all positive / negative random permutations R_p and R_n, as well as a number of random permutations N_R, the cluster *p*-values are calculated as:
$p(C_p) = (\ T(R_p) \geq T(C_p)\ )\ /\ N_R$
$p(C_n) = (\ T(R_n) \leq T(C_n)\ )\ /\ N_R$

H: Time course of overall LDT and induction task performance in Study 2

The observed effect course of the modulation of priming by cued task sets indicated a change in how participants prepare for the induction tasks / process the task cues, suggesting that the influence of task preparation for the induction tasks on the (intervening) LDT decreases, when they have more task practice later on during the course of the experiment. Regarding this postulated processing change, it may also be indicative how the overall performance in the LDT and the induction tasks changed during the course of the experiment. Therefore, we related the overall performance in the LDT (averaged across task set and semantic relatedness conditions), as well as the overall performance in induction tasks (averaged across task set conditions) to the effect course of the priming modulation by task sets for RTs (as described in the main text).

The following plots show the effect course of the priming modulation by cued task sets alongside with the time course of averaged RT / ER in the LDT and the induction tasks.

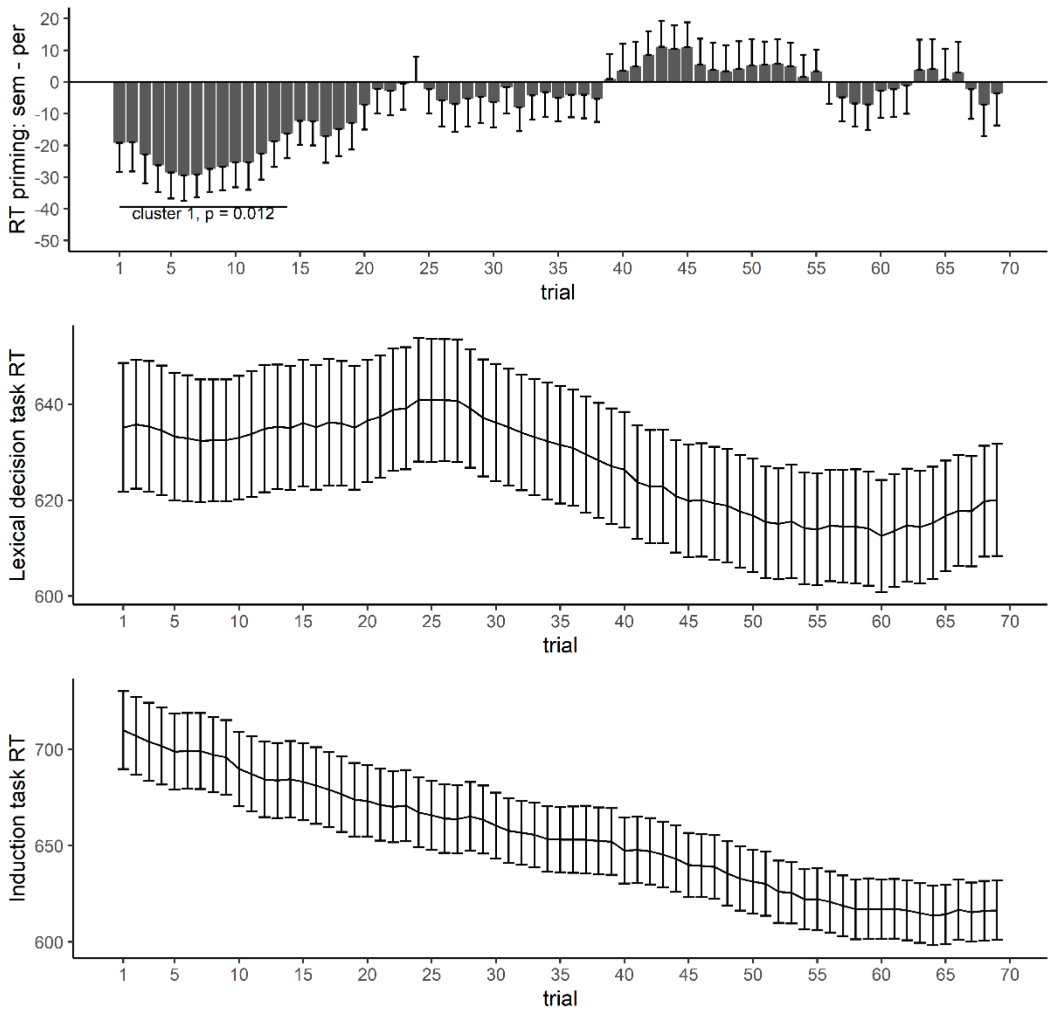

Figure S16 Time course of average RTs in the LDT (middle panel) and induction tasks (lower panel) alongside the effect course of the observed modulation of priming by cued task sets (upper panel).

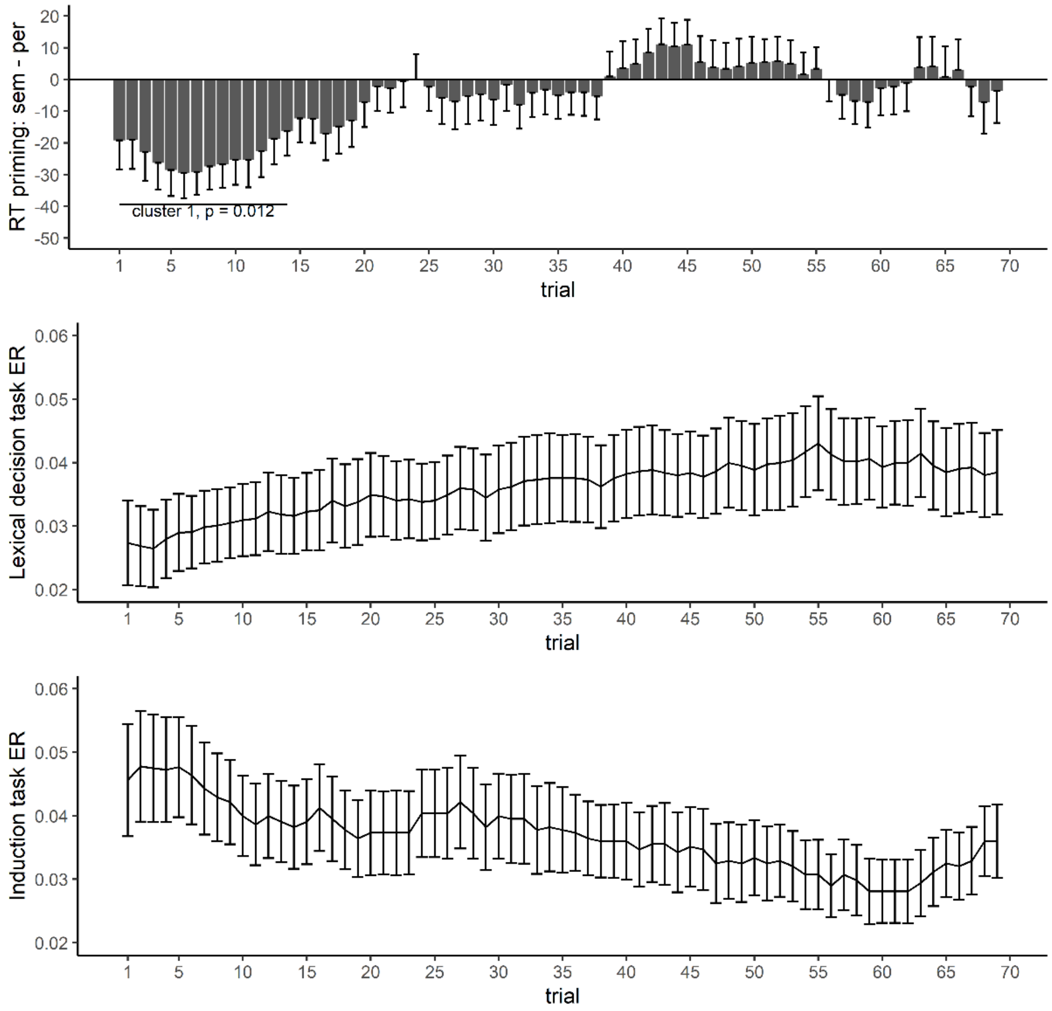

Figure S17 Time course of average ERs in the LDT (middle panel) and induction tasks (lower panel) alongside the effect course of the observed modulation of priming by cued task sets (upper panel).

These time courses indicated that for induction tasks, the performance progressively improved, indicated by continuously decreasing RTs and ERs during the course of the experiment. In contrast, for the LDT, RT performance was somehow stable at the beginning of the experiment and started only to improve after about a third of the experiment's duration. For ERs, performance in the LDT slightly decreased throughout the experiment (indicated by increased ERs).

Besides these descriptive patterns, we also tried to quantify the relation of the priming modulation with overall performance in the LDT and induction tasks. Therefore, we calculated the average Euclidean distance between the relevant curves with help of the respective default *R* function ("dist"), that is how similar the *shape* of the different curves were. For RTs, the average Euclidean distance was smaller between the curve of the priming modulation and the LDT performance (17.79) compared to between the priming modulation and induction task performance (36.45). For ERs, the average Euclidean distance was equal between priming – LDT curves (12.21) and priming – induction task curves (12.21).

Hence, these analyses indicated a larger similarity between the course of the priming modulation and the LDT (considering RTs). When inspecting *figure S16*, one can see that RTs in the LDT remained somehow stable until the postulated processing changes occurred (see the main text), i.e. until the priming modulation in the effect course analysis had vanished as well. Only afterwards, LDT performance started to improve. In contrast, for induction tasks, the performance improved continuously, showing no signs for a relation of the change in the course of overall induction task RTs / ERs with the effect course of the priming modulation. Accordingly, when comparing the overall performance in the LDT and induction tasks with the observed priming modulation, it seems likely that the postulated processing changes mainly affected the LDT, i.e. the task directly presented after the task cues (and the task where masked semantic priming was assessed).

I: Effect course of task set switch effects in induction tasks in Study 2

The observed effect course of the modulation of priming by cued task sets indicated a vanishing of that modulation, suggesting that only at the beginning of the experiment, the activation of task sets following task cue presentation was strong enough to accordingly sensitize subsequent priming. Besides this observed time course of the priming modulation, the strength of the activation of cued task sets could also be assessed (indirectly) via switch costs in induction tasks. A stronger task set activation likely would lead to larger interference if the other, conflicting task set is required in the following trial and henceforth should result in larger switch costs (Kiesel et al., 2010; Vandierendonck et al., 2010). Accordingly, one would expect to observe a decrease of task set switch costs in induction tasks throughout the experiment, as the modulation of priming by task sets indicated a stronger task set activation following task cue presentation at the beginning of the experiment. Hence, we tested how task set switch costs on RTs / ERs in induction tasks developed throughout the course of the experiment. Note that we included both only cued and executed task sets in these analyses, i.e. if the preceding trial was a task cue-only or an induction task one, to closely mimic the priming analyses. The priming analyses included both induction task trials and task cue-only trials, as the trial type was not evident until the end of the LDT and we therefore assumed the strength of activation of cued task sets to be similar in both trial types. Furthermore, note that the present study was not designed to investigate task set switch costs. For example, in contrast to typical experiments in task switching, there was a self-paced break at the end of each trial, where participants could prepare for the next trial. This additional preparation time likely decreased the influence of task set switch / repetition effects and the present analyses should therefore be taken with caution.

Effect course analyses for RTs in induction tasks depending on whether the task set switched or repeated from the previous trial revealed no significant cluster. However, corresponding analyses on ERs showed one significant cluster, $T = 53.03$, $p = .008$, trials 1 – 19, showing more errors if the task set switched compared to when it repeated from the previous trial, but only at the beginning of the experiment. This effect course showing a rapid vanishing of task set switch costs (compare *figure S18*) was therefore in line with the modulation of priming as described in the main experiment, suggesting a weaker influence of only cued or executed task sets with progressing experimental duration.

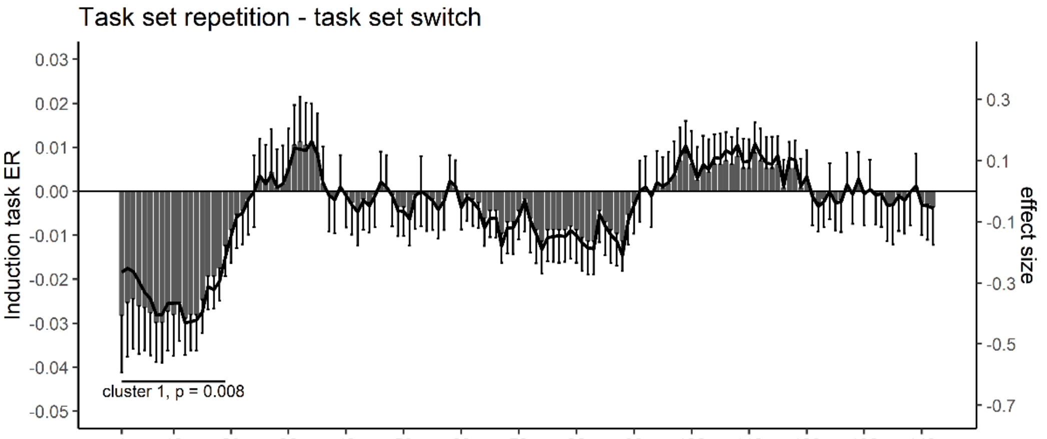

Figure S18 Effect course of the task set switch / repetition effect on ERs in induction tasks in Study 2. Negative values indicate larger ERs for task set switches (switch costs). Cluster $T = -53.03$, $p = .008$, trials 1 – 19.

J: Comparing the effect course analysis with a more traditional analysis approach separating the experiment into blocks

The effect course analysis in Study 2 revealed a modulation of masked priming by cued task sets, which however could only be observed at the beginning of the experiment and vanished rapidly. This interpretation of the effect change observed in Study 2 was grounded in the presence of a significant cluster at the beginning of the experiment and the later absence of any significant clusters. As the cluster-based permutation testing approach does not allow for a direct statistical comparisons of clusters (or a statistical comparison of clusters with other parts of the data not forming a cluster), such an interpretation therefore must rest on a descriptive inspection of the effect course. Only the pure presence of any differences (reflected by a significant cluster) can be statistically confirmed by the permutation test procedure (Maris & Oostenveld, 2007; Sassenhagen & Draschkow, 2019).

However, we think the implied relevance of a visual inspection of the time course of an effect by the proposed effect course analysis may even be helpful. While this method does not allow for a direct *statistical* test of the *change* of an effect, the effect course analysis allows for a continuous inspection of the time course of an effect, which might be useful in itself for a researcher. In contrast, while a more traditional analysis approach which splits the data into blocks and adds the block number as additional factor (into e.g., an ANOVA), allows for a direct statistical test of the change of an effect (interaction of the relevant effect with the block factor), it does not allow for a continuous inspection of the effect's time course. To elucidate this difference between both analysis approaches, we performed a traditional analysis approach of the modulation of priming by cued task sets in Study 2 by splitting the data into blocks and including the block number as additional factor into statistical analysis.

Therefore, we tested RTs in the LDT in Study 2 with a repeated-measures ANOVA depending on task set, semantic relatedness of prime and LDT target and experimental block and checked whether the interaction of task set, semantic relatedness and block reached significance, i.e. whether the ANOVA indicated a change in the modulation of priming by task sets. This leads to a critical caveat; the specification of the number of experimental blocks, which appears somehow arbitrary. To be able to more comprehensively elucidate this arbitrariness, we chose to test various block numbers with different numbers of trials included per block. We tested a number of 2 to 16 blocks, corresponding to a number of on average about 37 to 5 condition-wise trials included per block.

To summarize these analyses, overall (naturally) a comparable pattern as in the effect course analysis was observed, i.e. semantic priming was larger for semantic than perceptual task sets only at the beginning of the experiment. While some of the analyses with small block numbers revealed a significant change of the modulation of priming by cued task sets, i.e. a significant interaction, these analyses were (due to the larger block size) relatively uninformative about the duration and / or offset of this modulation. In contrast, analyses with smaller block sizes (i.e. more blocks), which had a higher temporal resolution, mostly failed to produce a significant interaction. Lastly, and most crucial in our perspective, the p-value of the interaction between task set, semantic relatedness and block largely differed between analyses with different block numbers and further showed no consistent pattern, i.e. it seemed somehow arbitrary which number of blocks produced a large or small p-value, see *figure S19*. For individual plots of the analyses with different block numbers, see the Open Science Framework (https://osf.io/6buz9/).

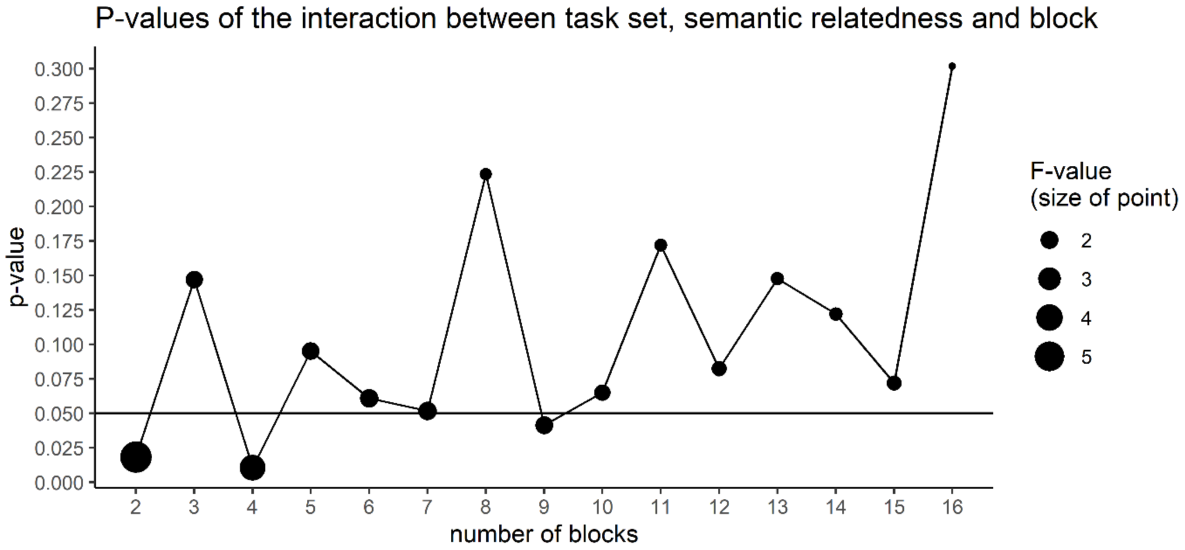

Figure S19 P-values for the interaction between task set, semantic relatedness and block in a traditional ANOVA analysis with various block numbers in Study 2. The analyzed interaction represents how the modulation of priming by cued task sets differed between blocks. i.e. a change of the modulation of priming by cued task sets. Most of the analyses failed to produce a significant effect. Moreover, there appears to be no clear pattern relating the number of blocks to the *p*-value of the interaction, rendering the choice of the block number somehow arbitrary. The horizontal line shows the typical alpha value, $\alpha = .05$.

Accordingly, the necessity for choosing an appropriate block number appears to be a disadvantage of the traditional analysis approach. Further, taking a factor with many levels (i.e. many blocks) into an ANOVA analysis may not be optimal and the power of such an interaction strongly depends on the shape of the interaction, i.e. how the effect (change) is distributed across blocks (cf. Sommet et al., 2023). The effect course analysis in contrast analyzes the data in a continuous manner. However, it also requires the definition of a window size, i.e. the amount of how strongly the single-trial data is smoothed (number of trials included in the moving averages). Hence, we also re-calculated the effect course analysis with various window sizes. Thus, we aim to demonstrate that the large variability observed in the block analyses is a consequence of the analysis approach, i.e. splitting the data into discrete blocks, while a continuous inspection of the time course of an effect with the effect course analysis reveals a more consistent pattern for varying temporal resolutions. Accordingly, we repeated the effect course analyses of Study 2 with window sizes corresponding to the same numbers of included trials as in the block analyses described above, i.e. window sizes from 5 to 37 (in steps of 2, as the effect course analysis requires odd window sizes). While this approach was expected to result in different shapes of the effect courses (too small window sizes lead to increased noise, while too large window sizes conceal fine-grained changes), we expected the presence / absence of clusters to remain more or less consistent across the different window sizes.

As expected, and in contrast to the analysis approach using blocks, the results of the effect course analyses were somehow comparable between the different chosen window sizes. All analyses revealed a significant cluster at the beginning of the experiment and there was a large consistency regarding the *p*-value of this cluster between the different window sizes, see *figure S20*. Accordingly, the effect course analysis showed a larger robustness concerning the chosen window size compared to the choice of the block number in the ANOVA analysis. However,

while all effect course analyses identified the location of this cluster to be at the beginning of the experiment, the shape of the individual effect courses differed as expected and the length of the cluster, i.e. the number of included trials, increased with larger window sizes. For the individual plots, see the Open Science Framework (https://osf.io/6buz9/). While such an increase in cluster length is an expectable property of the cluster-based permutation testing approach due to stronger smoothing with larger window sizes, the choice of an optimal window size remains an open question for further (methodological) studies.

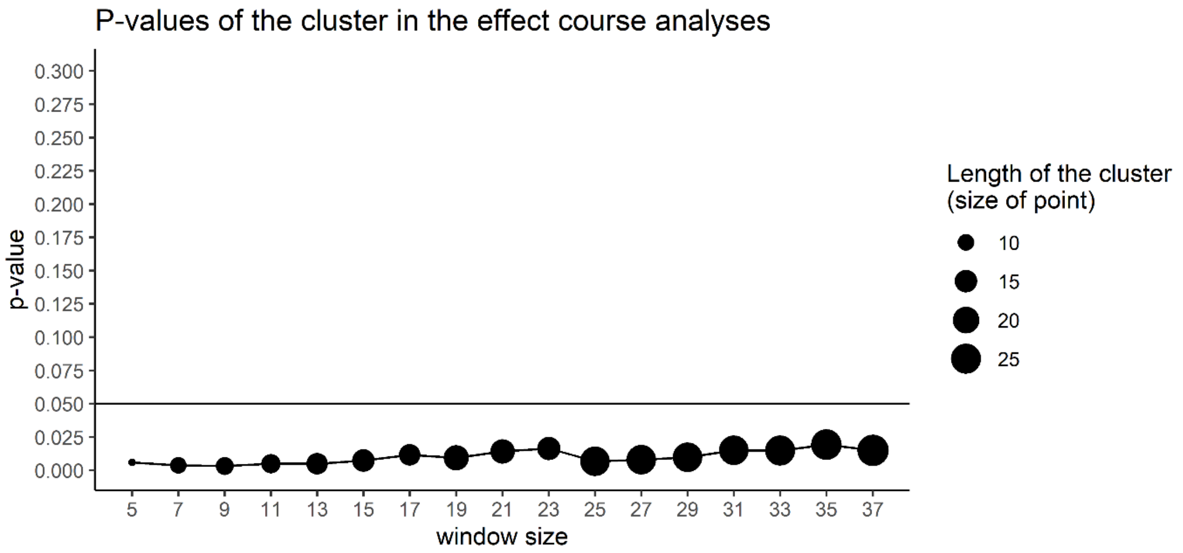

Figure S20 P-values for the cluster showing larger semantic priming for semantic compared to perceptual cued task sets at the beginning of Study 2 depending on the chosen window size for the effect course analysis. All analyses consistently revealed a significant cluster, and there were remarkable consistencies between window sizes (e.g. no large "jumps" of the p-value as a function of window size). The horizontal line shows the typical alpha value, α = .05.

K: Re-analysis of the effect course analysis in Study 2 depending on previous trial type and task set sequence

The usage of task cues was previously shown to depend on the validity of the task cue in the preceding trial, e.g. task cue-induced preparation was reduced if an invalid task cue was presented in the previous trial (Hübner et al., 2004; Wendt et al., 2012). Similarly, it may be possible that task cues are used differentially if the task set switches compared to when it repeats from the previous trial. In order to estimate the influence of such processes, we repeated the effect course analysis in Study 2 depending on previous trial type and task set sequence.

First, we calculated the effect course analysis separately for trials, which were preceded by an induction task trial, i.e. where the cued task set was indeed executed, and for trials, which were preceded by a task cue-only trial, i.e. where the task set was only cued, but not executed. According to an above outlined reduced usage of task cues, if the task cue in the preceding trial was invalid (in the present case: the cued task set was not executed), one would expect stronger usage of task cues following an induction task trial. Hence, the observed modulation of priming by cued task sets should be more pronounced following an induction task trial compared to a task cue-only trial. Note that as the data was split for these analyses according to the previous trial type, there were naturally only half of the trials available for the effect course analysis compared to the analysis reported in the main text. These effect course analyses (and all following analyses) were therefore performed with the same settings as described in the main text except for the window size. Due to only half of the trials remaining for the respective effect course analyses, we also divided the chosen window size by 2 to avoid any concealment of fine-grained changes. Hence, we took a window size of 9 for all following effect course analyses (40 / 5 + 1 = 9).

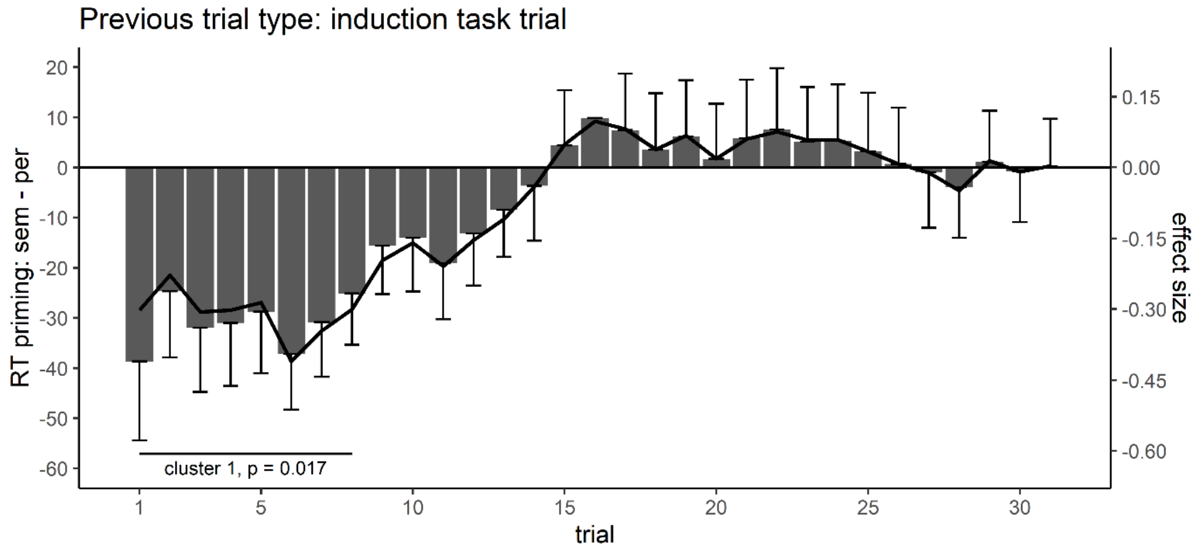

Figure S21 Effect course analysis of RTs in Study 2 for the modulation of priming by cued task sets, if the preceding trial was an induction task one. Negative values indicate larger semantic priming following a cued semantic task set. Cluster T = -20.29, p = .017, trials 1 – 8.

The effect course analysis on RTs revealed a significant cluster at the beginning of the experiment, if the preceding trial was an induction task trial, cluster T = -20.29, p = .017, trials

1 – 8, see *figure S21*. In contrast, if the previous trial was a task cue-only trial, no significant cluster could be observed. However, the overall course of the effect was similar compared to when the previous trial was an induction task trial, but less pronounced (see *figure S22*). While the overall pattern also indicated an initial modulation of priming by cued task sets when the previous trial was a task cue-only one (in terms of larger semantic priming for semantic task sets), this effect appeared to be too weak to form a significant cluster. Nevertheless, when contrasting the two effect courses, that is testing whether the difference between the effect courses depending on the previous trial type reached significance, a corresponding effect course analysis of the difference score revealed no significant cluster. For corresponding analyses on ERs, no significant cluster at all could be observed.

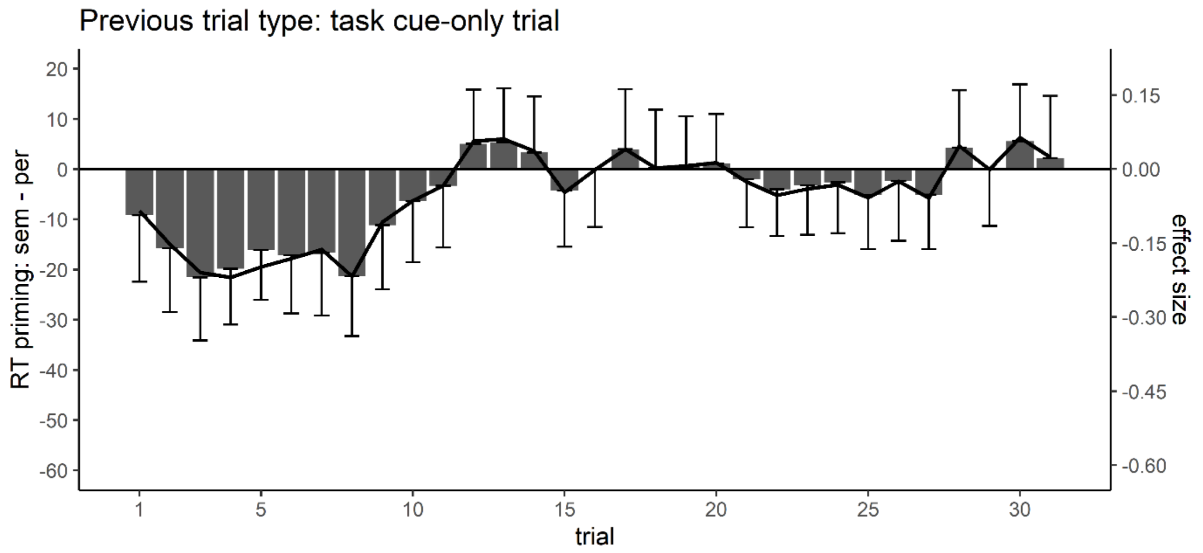

Figure S22 Effect course analysis of RTs in Study 2 for the modulation of priming by cued task sets, if the preceding trial was a task cue-only one. No significant cluster was observed.

Furthermore, we tested whether the modulation of priming by cued task sets as observed in Study 2 depended on the task set sequence, i.e. whether the (only cued or cued and executed) task set switched or repeated from the previous trial. With this regard, note that the present experiment was not designed to investigate task switching effects, see also *Supplementary Material I*. Nevertheless, to gauge the impact of such possible effects, we performed the effect course analysis in Study 2 separately for trials, where the task set repeated as well as for trials where the task set switched from the preceding trial.

For the analysis of RTs, while the overall time course of the effect was more or less comparable to the effect course analysis reported in the main text, the effect course analyses for trials where the task set was repeated, as well as for trials where the task set switched, did not reveal any significant cluster. Furthermore, for corresponding analyses on ERs, no significant cluster could be observed as well. For plots of all individual effect courses see the Open Science Framework (https://osf.io/6buz9/).

Supplementary references

Adelman, J. S., Johnson, R. L., McCormick, S. F., McKague, M., Kinoshita, S., Bowers, J. S., Perry, J. R., Lupker, S. J., Forster, K. I., Cortese, M. J., Scaltritti, M., Aschenbrenner, A. J., Coane, J. H., White, L., Yap, M. J., Davis, C., Kim, J., & Davis, C. J. (2014). A behavioral database for masked form priming. *Behavior Research Methods*, *46*(4), 1052–1067. https://doi.org/10.3758/S13428-013-0442-Y/TABLES/8

Bates, D., Mächler, M., Bolker, B. M., & Walker, S. C. (2014). Fitting Linear Mixed-Effects Models using lme4. *Journal of Statistical Software*, *67*(1). https://doi.org/10.18637/jss.v067.i01

Berger, A., & Kiefer, M. (2023). Electrophysiological correlates of response time outliers: Outlier related potentials. *Psychophysiology*, *60*(6), e14305. https://doi.org/10.1111/PSYP.14305

Berger, A., Kunde, W., & Kiefer, M. (2022). Task cue influences on lexical decision performance and masked semantic priming effects: The role of cue-task compatibility. *Attention, Perception, and Psychophysics*, *84*(8), 2684–2701. https://doi.org/10.3758/S13414-022-02568-2/FIGURES/3

Cousineau, D., & Chartier, S. (2010). Outliers detection and treatment: a review. *International Journal of Psychological Research*, *3*(1), 58–67. https://doi.org/10.21500/20112084.844

Hübner, M., Kluwe, R. H., Luna-Rodriguez, A., & Peters, A. (2004). Task preparation and stimulus-evoked competition. *Acta Psychologica*, *115*(2–3), 211–234. https://doi.org/10.1016/j.actpsy.2003.12.007

JASP Team. (2020). *JASP (Version 0.14.1)[Computer software]*. https://jasp-stats.org/

Kahveci, S., Rinck, M., Hannah Van Alebeek, ·, & Blechert, J. (2023). How pre-processing decisions affect the reliability and validity of the approach–avoidance task: Evidence from simulations and multiverse analyses with six datasets. *Behavior Research Methods 2023*, *1*, 1–32. https://doi.org/10.3758/S13428-023-02109-1

Kiefer, M. (2002). The N400 is modulated by unconsciously perceived masked words: Further evidence for an automatic spreading activation account of N400 priming effects. *Cognitive Brain Research*, *13*(1), 27–39. https://doi.org/10.1016/S0926-6410(01)00085-4

Kiefer, M., & Martens, U. (2010). Attentional sensitization of unconscious cognition: Task sets modulate subsequent masked semantic priming. *Journal of Experimental Psychology: General*, *139*(3), 464–489. https://doi.org/10.1037/a0019561

Kiesel, A., Steinhauser, M., Wendt, M., Falkenstein, M., Jost, K., Philipp, A. M., & Koch, I. (2010). Control and interference in task switching-A review. *Psychological Bulletin*, *136*(5), 849–874. https://doi.org/10.1037/a0019842

Kuznetsova, A., Brockhoff, P. B., & Christensen, R. H. B. (2017). lmerTest Package: Tests in Linear Mixed Effects Models. *Journal of Statistical Software*, *82*(13), 1–26. https://doi.org/10.18637/JSS.V082.I13

Maris, E., & Oostenveld, R. (2007). Nonparametric statistical testing of EEG- and MEG-data. *Journal of Neuroscience Methods*, *164*(1), 177–190. https://doi.org/10.1016/J.JNEUMETH.2007.03.024

Martens, U., Ansorge, U., & Kiefer, M. (2011). Controlling the unconscious: Attentional task sets modulate subliminal semantic and visuomotor processes differentially. *Psychological Science*, *22*(2), 282–291. https://doi.org/10.1177/0956797610397056

Miller, J. (2023). How Many Participants? How Many Trials? Maximizing the Power of Reaction Time Studies. *Behavior Research Methods*, 1–24. https://doi.org/10.3758/S13428-023-02155-9/FIGURES/17

Oostenveld, R., Fries, P., Maris, E., & Schoffelen, J. M. (2011). FieldTrip: Open source software for advanced analysis of MEG, EEG, and invasive electrophysiological data. *Computational Intelligence and Neuroscience*, *2011*. https://doi.org/10.1155/2011/156869

Pernet, C. R., Latinus, M., Nichols, T. E., & Rousselet, G. A. (2015). Cluster-based computational methods for mass univariate analyses of event-related brain potentials/fields: A simulation study. *Journal of Neuroscience Methods*, *250*, 85–93. https://doi.org/10.1016/J.JNEUMETH.2014.08.003

Ratcliff, R., & McKoon, G. (2008). The diffusion decision model: Theory and data for two-choice decision tasks. *Neural Computation*, *20*(4), 873–922. https://doi.org/10.1162/NECO.2008.12-06-420

Sassenhagen, J., & Draschkow, D. (2019). Cluster-based permutation tests of MEG/EEG data do not establish significance of effect latency or location. *Psychophysiology*, *56*(6), e13335.

https://doi.org/10.1111/PSYP.13335

Sommet, N., Weissman, D. L., Cheutin, N., & Elliot, A. J. (2023). How Many Participants Do I Need to Test an Interaction? Conducting an Appropriate Power Analysis and Achieving Sufficient Power to Detect an Interaction. *Advances in Methods and Practices in Psychological Science*, *6*(3). https://doi.org/10.1177/25152459231178728/ASSET/IMAGES/LARGE/10.1177_25152459231178728-FIG6.JPEG

Vandierendonck, A., Liefooghe, B., & Verbruggen, F. (2010). Task Switching: Interplay of Reconfiguration and Interference Control. *Psychological Bulletin*, *136*(4), 601–626. https://doi.org/10.1037/A0019791

Voss, A., Nagler, M., & Lerche, V. (2013). Diffusion models in experimental psychology: a practical introduction. *Experimental Psychology*, *60*(6), 385–402. https://doi.org/10.1027/1618-3169/A000218

Wagenmakers, E.-J., Love, J., Marsman, M., Jamil, T., Ly, A., Verhagen, J., Selker, R., Gronau, Q. F., Dropmann, D., Boutin, B., Meerhoff, F., Knight, P., Raj, A., van Kesteren, E.-J., van Doorn, J., Šmíra, M., Epskamp, S., Etz, A., Matzke, D., … Morey, R. D. (2017). Bayesian inference for psychology. Part II: Example applications with JASP. *Psychonomic Bulletin & Review 2017 25:1*, *25*(1), 58–76. https://doi.org/10.3758/S13423-017-1323-7

Wendt, M., Luna-Rodriguez, A., Reisenauer, R., Jacobsen, T., & Dreisbach, G. (2012). Sequential modulation of cue use in the task switching paradigm. *Frontiers in Psychology*, *3*, 287. https://doi.org/10.3389/fpsyg.2012.00287

Wiecki, T. V, Sofer, I., & Frank, M. J. (2013). HDDM: Hierarchical Bayesian estimation of the Drift-Diffusion Model in Python. *Frontiers in Neuroinformatics*, *0*(JULY 2013), 14. https://doi.org/10.3389/FNINF.2013.00014
